# Preterm Birth and Childhood Wheezing Disorders: A Systematic Review and Meta-Analysis

**DOI:** 10.1371/journal.pmed.1001596

**Published:** 2014-01-28

**Authors:** Jasper V. Been, Marlies J. Lugtenberg, Eline Smets, Constant P. van Schayck, Boris W. Kramer, Monique Mommers, Aziz Sheikh

**Affiliations:** 1 Department of Paediatrics, Maastricht University Medical Centre, Maastricht, Netherlands; 2 School for Public Health and Primary Care (CAPHRI), Maastricht University, Maastricht, Netherlands; 3 Allergy and Respiratory Research Group, Centre for Population Health Sciences, The University of Edinburgh, Edinburgh, United Kingdom; 4 School for Oncology and Developmental Biology (GROW), Maastricht University Medical Centre, Maastricht, Netherlands; 5 School of Mental Health and Neuroscience (MHeNS), Maastricht University Medical Centre, Maastricht, Netherlands; 6 Harkness Fellow in Health Care Policy and Practice, Division of General Internal Medicine and Primary Care, Brigham and Women's Hospital/Harvard Medical School, Boston, Massachusetts, United States of America; Simon Fraser University, Canada

## Abstract

**Background:**

Accumulating evidence implicates early life factors in the aetiology of non-communicable diseases, including asthma/wheezing disorders. We undertook a systematic review investigating risks of asthma/wheezing disorders in children born preterm, including the increasing numbers who, as a result of advances in neonatal care, now survive very preterm birth.

**Methods and Findings:**

Two reviewers independently searched seven online databases for contemporaneous (1 January 1995–23 September 2013) epidemiological studies investigating the association between preterm birth and asthma/wheezing disorders. Additional studies were identified through reference and citation searches, and contacting international experts. Quality appraisal was undertaken using the Effective Public Health Practice Project instrument. We pooled unadjusted and adjusted effect estimates using random-effects meta-analysis, investigated “dose–response” associations, and undertook subgroup, sensitivity, and meta-regression analyses to assess the robustness of associations.

We identified 42 eligible studies from six continents. Twelve were excluded for population overlap, leaving 30 unique studies involving 1,543,639 children. Preterm birth was associated with an increased risk of wheezing disorders in unadjusted (13.7% versus 8.3%; odds ratio [OR] 1.71, 95% CI 1.57–1.87; 26 studies including 1,500,916 children) and adjusted analyses (OR 1.46, 95% CI 1.29–1.65; 17 studies including 874,710 children). The risk was particularly high among children born very preterm (<32 wk gestation; unadjusted: OR 3.00, 95% CI 2.61–3.44; adjusted: OR 2.81, 95% CI 2.55–3.12). Findings were most pronounced for studies with low risk of bias and were consistent across sensitivity analyses. The estimated population-attributable risk of preterm birth for childhood wheezing disorders was ≥3.1%.

Key limitations related to the paucity of data from low- and middle-income countries, and risk of residual confounding.

**Conclusions:**

There is compelling evidence that preterm birth—particularly very preterm birth—increases the risk of asthma. Given the projected global increases in children surviving preterm births, research now needs to focus on understanding underlying mechanisms, and then to translate these insights into the development of preventive interventions.

**Review Registration:**

PROSPERO CRD42013004965

*Please see later in the article for the Editors' Summary*

## Abstract 2

In a systematic review and meta-analysis, Jasper Been and colleagues investigate the association between preterm birth and the development of wheezing disorders in childhood.

*Please see later in the article for the Editors' Summary*

## Editors' Summary

**Background:**

Most pregnancies last around 40 weeks, but worldwide, more than 11% of babies are born before 37 weeks of gestation (the period during which a baby develops in its mother's womb). Preterm birth is a major cause of infant death—more than 1 million babies die annually from preterm birth complications—and the number of preterm births is increasing globally. Multiple pregnancies, infections, and chronic (long-term) maternal conditions such as diabetes can all cause premature birth, but the cause of many preterm births is unknown. The most obvious immediate complication that is associated with preterm birth is respiratory distress syndrome. This breathing problem, which is more common in early preterm babies than in near-term babies, occurs because the lungs of premature babies are structurally immature and lack pulmonary surfactant, a unique mixture of lipids and proteins that coats the inner lining of the lungs and helps to prevent the collapse of the small air sacs in the lungs that absorb oxygen from the air. Consequently, preterm babies often need help with their breathing and oxygen supplementation.

**Why Was This Study Done?:**

Improvements in the management of prematurity mean that more preterm babies survive today than in the past. However, accumulating evidence suggests that early life events are involved in the subsequent development of non-communicable diseases (non-infectious chronic diseases). Given the increasing burden of preterm birth, a better understanding of the long-term effects of preterm birth is essential. Here, the researchers investigate the risks of asthma and wheezing disorders in children who are born preterm by undertaking a systematic review (a study that uses predefined criteria to identify all the research on a given topic) and a meta-analysis (a statistical method for combining the results of several studies). Asthma is a chronic condition that is caused by inflammation of the airways. In people with asthma, the airways can react very strongly to allergens such as animal fur and to irritants such as cigarette smoke. Exercise, cold air, and infections can also trigger asthma attacks, which can sometimes be fatal. The symptoms of asthma include wheezing (a high-pitched whistling sound during breathing), coughing, chest tightness, and shortness of breath. Asthma cannot be cured, but drugs can relieve its symptoms and prevent acute asthma attacks.

**What Did the Researchers Do and Find?:**

The researchers identified 30 studies undertaken between 1995 and the present (a time span chosen to allow for recent changes in the management of prematurity) that investigated the association between preterm birth and asthma/wheezing disorders in more than 1.5 million children. Across the studies, 13.7% of preterm babies developed asthma/wheezing disorders during childhood, compared to only 8.3% of babies born at term. Thus, the risk of preterm babies developing asthma or a wheezing disorder during childhood was 1.71 times higher than the risk of term babies developing these conditions (an unadjusted odds ratio [OR] of 1.71). In analyses that allowed for confounding factors—other factors that affect the risk of developing asthma/wheezing disorders such as maternal smoking—the risk of preterm babies developing asthma or a wheezing disorder during childhood was 1.46 times higher than that of babies born at term (an adjusted OR of 1.46). Notably, compared to children born at term, children born very early (before 32 weeks of gestation) had about three times the risk of developing asthma/wheezing disorders in unadjusted and adjusted analyses. Finally, the population-attributable risk of preterm birth for childhood wheezing disorders was more than 3.1%. That is, if no preterm births had occurred, there would have been more than a 3.1% reduction in childhood wheezing disorders.

**What Do These Findings Mean?:**

These findings strongly suggest that preterm birth increases the risk of asthma and wheezing disorders during childhood and that the risk of asthma/wheezing disorders increases as the degree of prematurity increases. The accuracy of these findings may be affected, however, by residual confounding. That is, preterm children may share other, unknown characteristics that increase their risk of developing asthma/wheezing disorders. Moreover, the generalizability of these findings is limited by the lack of data from low- and middle-income countries. However, given the projected global increases in children surviving preterm births, these findings highlight the need to undertake research into the mechanisms underlying the association between preterm birth and asthma/wheezing disorders and the need to develop appropriate preventative and therapeutic measures.

**Additional Information:**

Please access these websites via the online version of this summary at http://dx.doi.org/10.1371/journal.pmed.1001596.

- The March of Dimes, a nonprofit organization for pregnancy and baby health, provides information on preterm birth (in English and Spanish)
- Nemours, another nonprofit organization for child health, also provides information (in English and Spanish) on premature babies and on asthma (including personal stories)
- The UK National Health Service Choices website provides information about premature labor and birth and a real story about having a preterm baby; it provides information about asthma in children (including real stories)
- The MedlinePlus Encyclopedia has pages on preterm birth, asthma, asthma in children, and wheezing (in English and Spanish); MedlinePlus provides links to further information on premature birth, asthma, and asthma in children (in English and Spanish)

## Introduction

The impact of early life exposures on subsequent health and disease is increasingly recognised [1]. Preterm birth is a common early life event, the adverse consequences of which can affect the entire life course. Worldwide, over 11% of babies are born preterm, a number that continues to rise in most regions [2]. Prevention of preterm birth is currently limited by a knowledge gap regarding the mechanisms of normal and pathological labour onset [3]. Given the increasing burden of preterm birth and the restricted scope for prevention, a focus on appreciating and containing its consequences is warranted.

Respiratory distress syndrome is the most obvious direct manifestation of immaturity in preterm newborns. The combination of structural lung immaturity and pulmonary surfactant deficiency results in regional atelectasis and a varying degree of respiratory compromise [4]. Ventilatory support and additional oxygen supplementation are common necessities, and many very preterm babies go on to develop chronic lung disease (bronchopulmonary dysplasia [BPD]) [5]. The majority of preterm babies are, however, born close to term, when respiratory compromise is a less common event. Whereas late preterm babies have long been regarded a normal variation of term babies, it is increasingly recognised that they are at risk of a range of adverse outcomes, including respiratory disease [6].

Accumulating evidence now implicates preterm birth in the development of respiratory disease in later life. Small airway obstruction is evident through decreased forced expiratory volume in 1 s (FEV1) in children and adults born preterm [7], and a hypothesized link with chronic obstructive pulmonary disease in adulthood was recently confirmed [8]. Along this line of evidence, a meta-analysis published in 2006 identified preterm birth as a risk factor for asthma in a mixed paediatric and adult population [9]. Asthma is the most common chronic disease affecting children, and its link with preterm birth is of significant public health relevance given the increasing incidence of both entities [2],[10]. Of note, the majority of cohorts aggregated in this meta-analysis were born before the 1990s [9]. Important changes in neonatal clinical management have been introduced since, including increased use of antenatal steroids and postnatal surfactant, and a shift towards less aggressive respiratory management [4]. These changes have selectively impacted the survival of extremely preterm infants, and shifted the occurrence of acute respiratory problems towards lower gestational age groups [4]. Associated changes in long-term pulmonary outcomes may have occurred, and there is therefore a need for a comprehensive, rigorous assessment of the contemporaneous evidence base.

We aimed to investigate the association between preterm birth and childhood wheezing disorders through a systematic review and meta-analysis of studies with populations born from the 1990s onwards. We focused on wheezing disorders in general rather than just asthma, given the difficulty in differentiating between the two, particularly among young children [11]. Unlike previous work [9], we also considered the potential impact of confounding factors and explored the association by degree of prematurity. Based on aggregated association measures, we have furthermore estimated population-attributable risks (PARs) to quantify associated disease burden.

## Methods

### Search Strategy

This review was performed following the methods detailed in a systematic review protocol registered with PROSPERO (CRD42013004965; Text S1). Online databases were searched independently by two authors using the following search terms: PubMed and Embase: (preterm OR prematur*) AND (asthma OR wheez*); Google Scholar: asthma, wheeze, wheezing, preterm, premature, child, children, childhood; World Health Organization Global Health Library, World Health Organization Library Information System, SciELO, and Trip [12]: ((preterm OR premature OR preterms OR prematurity) AND (asthma OR wheeze OR wheezing)). The searches covered the period from 1 January 1995 to 23 September 2013. No language restrictions were applied. Additional studies were identified by screening reference lists of articles of interest and tracing citations of articles through ISI Web of Knowledge. We asked an international panel of experts in the field (Text S2) to report any additional published, unpublished, or in progress studies that might have been missed.

### Study Selection

Epidemiological studies were eligible for inclusion if they reported an association between preterm birth (<37 wk versus ≥37 wk gestation [term]) and asthma or wheezing in children (aged 0.5 to 18 y). Studies reporting on populations that included children born before 1995 were eligible for inclusion only when at least 50% of the cohort was born from 1995 onwards and none of the children was born before 1990, so as to primarily include studies conducted in the period following the important changes in neonatal practice outlined earlier. When studies with overlapping data were identified, the most informative study was selected for inclusion in this report; the determination of which study was considered most informative was based on consensus, study size being an important determinant. Final study selection was based on a consensus decision between reviewers, with arbitration by a third reviewer in case of disagreement.

### Data Extraction

Data were independently extracted from eligible studies by two reviewers (J. V. B. and M. J. L.); disagreements were resolved through discussion, with arbitration by a third reviewer if necessary (A. S.). The following study characteristics were extracted: authors, full reference, study design, location, sample size, inclusion and exclusion criteria, age range and birth year of study participants, method of ascertainment of exposure and outcomes, and outcome measure.

Post-term (>42 wk gestation) births were excluded from the analyses, if possible. If studies reported follow-up at different time points, the most recent was selected. Similarly to previous work [13], we used only one outcome measure from each study in the analyses; for studies with multiple outcome measures, the following hierarchy was used to select the outcome measure used in the analyses, from most preferred to least preferred: asthma, persistent wheezing, recurrent wheezing, severe wheezing, and wheezing. Furthermore, we used the following hierarchy to select the highest ascertainment level if the outcome was measured multiple ways: clinician diagnosis, documented medication use as a wheezing disorder proxy, routinely collected health-care data, parent- or patient-reported clinician diagnosis, parent- or patient-reported medication use, and parent- or patient-reported symptoms. “Ever” wheezing or asthma was favoured over recent and current wheezing or asthma.

Absolute patient counts were extracted to compose 2×2 tables according to preterm birth and wheezing disorder status, and these were used to calculate univariate odds ratios (ORs); in instances where relevant crude data were missing, we approached authors to request these. If needed, count data were calculated from provided percentages, and these were then rounded off to the nearest integer. Adjusted association measures were extracted from the most adjusted model presented.

### Study Quality

Study quality was assessed independently by two investigators using the Effective Public Health Practice Project quality assessment tool for quantitative studies [14]. Sex, maternal smoking during pregnancy, and maternal atopy or asthma, or a family history of atopy or asthma, were deemed the most important confounders. Any disagreement was resolved by consensus and arbitration by a third reviewer, where necessary.

### Statistical Analyses

Univariate association measures were pooled using Mantel-Haenszel analysis. Random-effects models were applied because of anticipated heterogeneity given between-country variation in clinical practice and differences in inclusion and exclusion criteria, outcome specification, and ascertainment level of exposure and outcome. Adjusted association measures were pooled via random-effects generic inverse variance analysis. Standard errors for study point estimates were calculated from the respective 95% confidence intervals as described in the Cochrane Handbook [15].

For studies reporting adjusted association measures according to multiple gestational age strata, the least preterm stratum was selected to obtain the most conservative estimate. In a separate analysis, a linear association between gestational age at birth and wheezing disorder risk was investigated, pooling individual studies by random-effects generic inverse variance analysis. For this purpose, adjusted association measures for multiple gestational age strata were aggregated within studies using a fixed-effects log-linear dose–response regression model [16]. The *Q*-statistic and *I* ^2^-test were used to assess heterogeneity among studies. Small-study effects were assessed using funnel plots and Harbord's modified regression test for unadjusted data and Egger's regression test for adjusted association measures.

### Subgroup Analyses

Subgroup analyses were performed according to age group (<5 y versus ≥5 y) and degree of prematurity (<32 wk versus 32–36 wk). When studies reported the association between preterm birth and wheezing disorders for both subgroups (i.e., follow-up at <5 y and at ≥5 y, and/or reported for <32 wk and 32–36 wk gestation), the corresponding association measures were included in both sides of the comparison.

### Sensitivity Analyses

Sensitivity analyses were performed according to risk of bias (low, moderate, or high), study size (*n*<10,000 versus *n*≥10,000), and outcome definition (asthma versus wheezing) and ascertainment (clinician diagnosis and/or medication use versus parent-reported outcomes).

### Meta-Regression Analysis

Meta-regression analysis was performed to assess the independent effects of study size, mean age, wheezing type (wheezing versus asthma), diagnosis ascertainment, and publication year on the association between preterm birth and wheezing disorders.

### Population-Attributable Risk

PAR was calculated using the following formula:
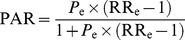
(1)where *P*_e_ is the exposure prevalence and RR_e_ is the relative risk due to exposure [17]. RR_e_ was calculated as follows:
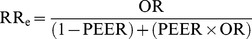
(2)where PEER is the patient expected event rate. As estimations of PAR based on unadjusted association measures can be biased by confounding of the exposure–outcome relationship, we additionally calculated PAR using aggregated adjusted association measures via the following formula:
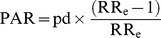
(3)where pd is the proportion of cases exposed [17].

Analyses were performed using Stata 12 (StataCorp).

### Role of the Funding Source

The funders had no role in study design, collection, analysis, interpretation of the data, writing of the report, or decision to submit the work for publication.

## Results

**Figure 1:**
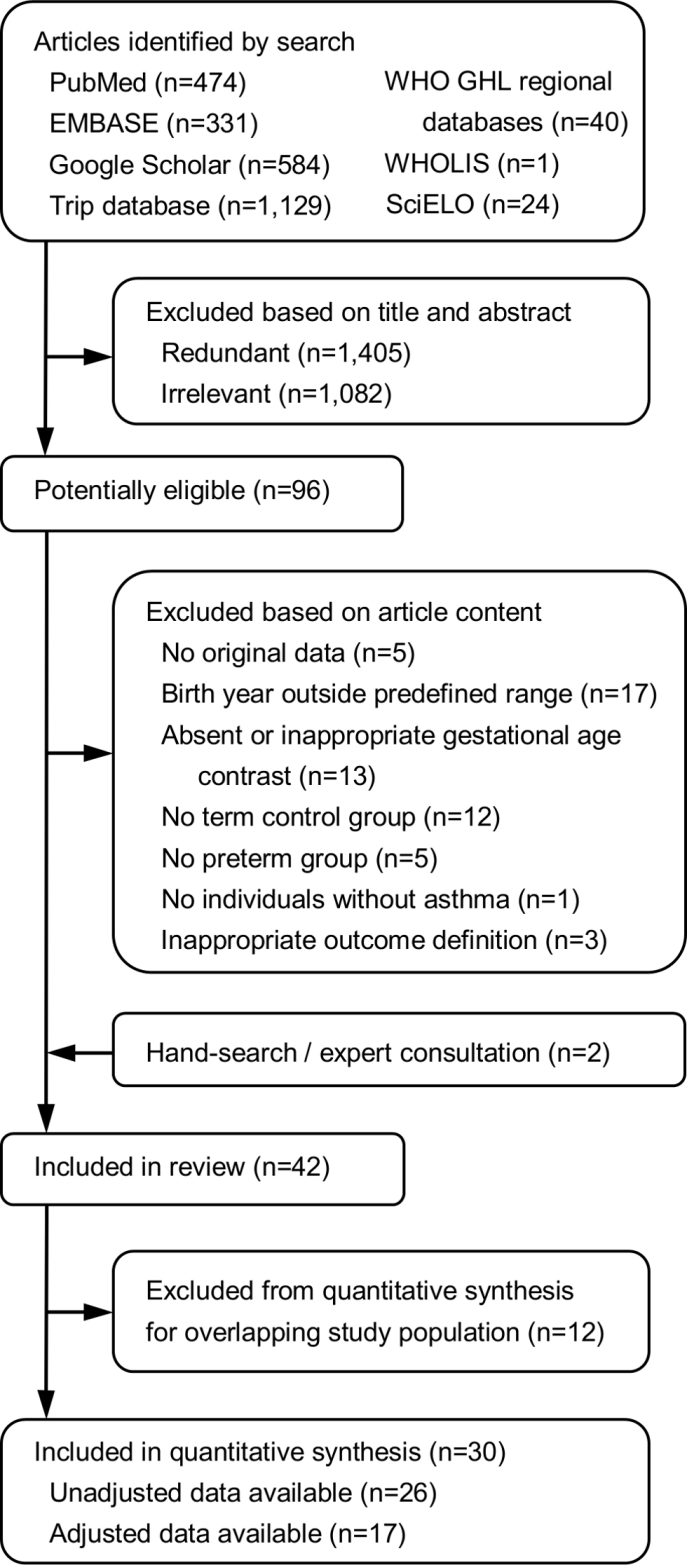
Flowchart outlining study selection. WHO GHL, World Health Organization Global Health Library; WHOLIS, World Health Organization Library Information System.

Forty-two eligible studies were identified [18]–[59]. Study selection is summarised in Figure 1. Five studies from Sweden [18],[20],[36],[47],[55] and two from northern California, US, had significant population overlap [28],[56]. Furthermore, several eligible studies described the same cohort: the Epicure cohort, UK and Ireland (*n* = 4) [29],[34],[41],[49]; the Boston Birth Cohort, Boston, US (*n* = 3) [39],[40],[44]; a birth cohort from southern New England, US (*n* = 2) [27],[37]; and cross-sectional surveys from Liverpool, UK (*n* = 2) [38],[53]. The most relevant study was selected from each set of overlapping studies. Characteristics of the 12 studies that were excluded, as well as reasons for exclusion, are given in Table S1. Meta-analysis was performed on the remaining 30 unique studies (Tables 1 and 2), which reported on a total of 1,543,639 individuals [19],[21]–[27],[29]–[33],[35],[36],[42]–[46],[48],[50]–[54],[56]–[59].

**Table 1:** Characteristics of included studies: cohort studies.

Study	Study Design	Sample Size	Inclusion Criteria	Exclusion Criteria	Birth Year	Country (Region)	Age at Follow-Up	Exposure Ascertainment	Outcome Definition Used in Meta-Analysis	Outcome Ascertainment	Risk of Bias^a^
Yuan 2003 [51]	RC	8,858	Born in North Jutland	None mentioned	1996–1997	Denmark (North Jutland)	1 y	Birth registry (LMP, adjusted by ultrasound if necessary)	Asthma drug prescription (β-agonist and inhaled glucocorticoid)	ATC code R03 in Pharmacoepidemiological Prescription Database of North Jutland	Low
Taveras 2006 [45]	PC	763	Singletons born after antenatal care in eight Harvard Vanguard Medical Associates offices	Consent withdrawn, unable to speak English, GA<34 wk	1999–2002	US (eastern Massachusetts)	2 y	Medical records (LMP/second trimester ultrasound when unreliable)	Asthma in first 2 y	Parent-reported physician diagnosis	High
Gessner 2007 [30]	RC	24,979	Children <10 y enrolled in Medicaid for >1 y	No match with birth certificate, incomplete data	1994–2001	US (Alaska)	1–4 y	Birth certificates (LMP)	Asthma	Asthma drug prescription and ICD-9 code 493.xx in Medicaid claims	Moderate
Yang 2007 [50]	PC	813	Born at Chang Gung Memorial Hospital	Emergency delivery, no follow-up	1999–2004	Taiwan (Kaoshiung)	1.5 y	Unclear	Frequent wheezing (≥4 episodes)	Parental questionnaire	Moderate
Castro-Rodriguez 2010 [24]	PC	1,409	Spanish children attending primary care centre in area	No contact data, no consent, incomplete data	2006–2007	Spain (Cartagena)	15–18 mo	Unclear	Wheezing in first year	Parental questionnaire	Moderate
Getahun 2010 [31]	RC	397,852	Singletons born alive in KPSC hospitals and enrolled as KPSC health plan members within 60 d of birth	GA <23 wk, birth defects, neonatal death	1991–2007	US (southern California)	<8 y	KPSC hospital records (LMP or clinical estimate)	Asthma	ICD-9-Clinical Modification code 493.xx and ≥2 asthma drug prescriptions in KPSC hospital records	Moderate
Rautava 2010 [43]	RC	1,299	Born at <32 wk or <1,500 g in level II–III hospital in Finland; sex-matched term babies	No personal identifier, missing/unlikely data, death <5 y, address abroad/unknown	2001–2002	Finland	5 y	National Medical Birth Register	Asthma	ICD-10 code J45 in National Hospital Discharge Register	High
Algert 2011 [52]	RC	240,511	Singletons surviving ≥2 y	Birth records with missing data	2001–2003	Australia (New South Wales)	2–5 y	Birth records (Midwives Data Collection)	Asthma-related hospital admission	ICD-10 code J45/46 in routinely collected data (Admitted Patient Data Collection)	Moderate
Álvarez 2011 [19]	RC	119	Born in University Clinical Hospital of Valladolid	No consent	1996–2001	Spain (Valladolid)	5–10 y	Unclear	Recurrent wheezing at school age	Parental questionnaire	High
Goyal 2011 [33]	RC	7,925	Under well-child care of Children's Hospital of Philadelphia Pediatric Research Consortium from within 30 d of birth to 18 mo	GA<34 or >42 wk, major congenital anomalies, hereditary disorders	2007	US (Philadelphia area)	≤1.5 y	Birth hospital discharge records (missing data imputed)	Asthma	ICD-9 code 493.xx in practice records	High
Bérard 2012 [21]	RC	36,055	Enrolled for at least 3 y in regional insurance (Régie de l'Assurance Maladie du Québec)	None mentioned	1997–2000	Canada (Québec)	3 y	Birth records (MED-ECHO)	Asthma diagnosis in hospital records (MED-ECHO)	ICD-9 code 493	Moderate
Boyle 2012 [22]	RC	14,273 at 3 y; 13,942 at 5 y	Sample of children born in England/Wales Sep 2000–Aug 2001 or in Scotland/Northern Ireland Nov 2000–Jan 2002 and alive and living in UK at 9 mo	Recent or temporary immigrants, missing or implausible GA	2000–2002	UK	9 mo, 3 y, 5 y	Maternal report of expected due date	Wheezing or whistling in chest in previous year	Parental questionnaire	Moderate
Robison 2012 [44]	PC	1,448	Singletons born at Boston Medical Center	No consent, incomplete data	1998–20	US (Boston)	0.5–6 y	Boston Medical Center hospital records (LMP and first trimester ultrasound)	Recurrent (≥4 episodes) wheezing	Physician-documented wheezing in Boston Medical Center medical records	Low
Sonnen-schein-van der Voort 2012 [54]	PC	5,125	Singleton children born to mothers in selected area of Rotterdam	No consent, no follow-up, born outside follow-up area, subsequent children in same mother, missing birth weight/asthma symptoms	2002–2006	Netherlands (Rotterdam)	1–4 y	Hospital and midwife records	Wheezing	Parental questionnaire	Low
Collier 2013 [27]	PC	1,428	Singletons from cohort enriched with asthmatic mothers	Non-English-speaking mothers, infant death, no consent, no medical record	1997–2000	US (New England)	6 y (±3 mo)	Unclear	Asthma and wheeze in prior year	Parent-reported physician diagnosis (asthma) or symptom (wheezing)	High
Escobar 2013 [56]	RC	72,602	In KPNC database and Kaiser Foundation Health Plan membership first 5 y	In-hospital death, GA<32 wk, incomplete data	1996–2004	US (northern California)	5 y	KPNC database	Asthma^b^ (unadjusted analysis); recurrent wheeze in fifth year after birth (adjusted analysis)	ICD-9 code 493.xx or 786.07, or asthma drug prescription in KPMCP hospital records	Moderate
Källén 2013 [36]	RC	708,907	Born in Sweden	Neonatal death, unknown GA	1994–20	Sweden	2–11 y	Swedish Medical Birth Register (first trimester ultrasound or LMP)	Asthma drug prescription (≥5 occasions)	ATC code R03 in Swedish Prescribed Drug Register	Low
Vrijlandt 2013 [48]	PC	2,111 at 4 y; 1,523 at 5 y	Preterm babies attending Preventive Child Health Care centre and very preterm babies from neonatal intensive care units; random term controls	Non-response, no consent	2001–2003	Netherlands	4 y; 5 y	Parental questionnaire/medical records	β-agonist ± inhaled glucocorticoid use (4 y); asthma (5 y)	Parental questionnaire	High

a Please see Table S2 for detailed assessment.

b ≥3 patient encounters ≥14 d apart, with a diagnosis of asthma or wheezing; and/or ≥1 such encounter with a prescription for oral corticosteroids between 2 d before and 7 d after the encounter; and/or ≥1 hospitalisations for ≥24 h or until death occurred, with a diagnosis of asthma or wheezing; and/or ≥4 dispensing events ≥14 d apart, for which selected asthma medications were prescribed plus ≥1 encounters with a diagnosis of asthma or wheezing; and/or death outside the hospital due to asthma or wheezing.

ATC, Anatomical Therapeutic Chemical Classification System; GA, gestational age; ICD, International Classification of Diseases; KPNC, Kaiser Permanente Northern California; KPSC, Kaiser Permanente Southern California; LMP, last menstrual period; PC, prospective cohort; RC, retrospective cohort.

**Table 2:** Characteristics of included studies: case control and cross-sectional studies.

Study	Study Design	Sample Size	Inclusion Criteria	Exclusion Criteria	Birth Year	Country (Region)	Age at Follow-Up	Exposure Ascertainment	Outcome Definition Used in Meta-Analysis	Outcome Ascertainment	Risk of Bias^a^
Gorman 2005 [32]	CS	1,173	Children born to Puerto-Rican women, oversampling of low birth weight babies	Non-response	1994–1995	US (Florida, Connecticut, Massachusetts, New Jersey, New York, Pennsylvania, Puerto Rico)	22 mo	Unclear	Ever asthma	Parent-reported health-care-provider diagnosis	High
Koshy 2010 [53]	CS	933	Children attending primary school in Merseyside	Non-response	1995–2001	UK (Merseyside)	5–11 y	Parental questionnaire	Asthma	Parent-reported physician diagnosis	High
Visser 2010 [46]	CS	1,115	Babies attending 13-mo well baby clinic	No consent	2004–2006	Netherlands (Zwolle area)	13 mo	Unclear	Recurrent (≥4 episodes) wheezing in first year	Parental questionnaire	Moderate
Civelek 2011 [26]	CS	6,219	Random sample of fifth grade students in five cities	None mentioned	1995–1996	Turkey (Ankara, Antalya, Manisa, Trabzon, Van)	10–11 y	Unclear	Recurrent (≥4 episodes) wheezing or whistling in chest in prior year	Parental questionnaire	Moderate
Fawke 2010 [29]	CC	343	GA<25 wk; matched term classmate controls	Lost to follow-up	1995	UK, Ireland	11 y	Hospital records	Asthma medication/wheeze in prior year and doctor-diagnosed asthma, or asthma medication and wheeze in prior year	Parental questionnaire	High
Herrera 2011 [58]	CS	678	Living in study area for >1 y	Chronic NDI/cardiac disease, no consent	2003–2009	Colombia (Bucaramanga)	<7 y	Parental questionnaire	>50% probability of asthma	Parental questionnaire	High
Brehm 2012 [23]	CC	560	Random sample of 6- to 14-y-olds in San Juan ± asthma	No consent, ≥1 non-Puerto Rican grandparent	1995–2004	Puerto Rico (San Juan)	6–14 y	Unclear	Asthma and wheeze in the prior year	Parent-reported physician diagnosis (asthma)/symptom (wheezing)	Moderate
Cheraghi 2012 [25]	CS	3,909	Random sample of school-attending children in Pune	Incomplete data, no consent	1994–1996; 2001–2003	India (Pune)	6–7, 13–14 y	Unclear	Ever asthma or wheezing in last year	Parental questionnaire	Moderate
Fauroux 2013 [57]	CC	443	GA<33 wk; matched term newborns born in level 2/3 unit	BPD, respiratory syncytial virus prophylaxis, non-French-speaking parents, no consent, serious chronic illness	2008–2009	France	1 y	Hospital records	Recurrent (>1 episode) wheezing in first year	Parental questionnaire	High
Joshi 2013 [35]	CS	90	GA≤32 wk ± BPD; term controls	Congenital anomalies, cardiopulmonary defect, NDI, non-compliance	1996–2001	UK	8–12 y	Hospital records	Asthma	Parent-reported physician diagnosis	High
Miyake 2013 [42]	CS	2,004	Attending physical examination at seven public health centres	No consent, missing data	2003–2004	Japan (Fukuoka)	3 y	Parental report from *Maternal and Child Health Handbook*	Ever asthma	Parental questionnaire	High
Nantanda 2013 [59]	CS	614	Presenting at emergency unit with cough and/or dyspnoea and tachypnoea	No consent, cardiac problems	2006–2012	Uganda (Kampala)	0–5 y	Unclear	Asthma^b^	Post hoc doctor diagnosis based on written records	High

a Please see Table S2 for detailed assessment.

b High probability of asthma defined as ≥4 out of the following five items: (1) ≥1 of the following: cough, wheeze, or difficulty breathing; (2) ≥1 of the following: recurrent cough, wheeze, and/or difficulty breathing; atopic history in child; history of asthma in first-degree relative; (3) ≥3 of the following: fast breathing, chest indrawing, prolonged expiration, or rhonchi; (4) good response to bronchodilators; (5) chest X-ray normal or showing hyperinflation.

CC, case control; CS, cross-sectional; GA, gestational age; NDI, neurodevelopmental impairment.

### Study Quality

Four studies were deemed to have low risk of bias, 12 had moderate risk of bias, and 14 had high risk of bias (Table S2). Studies with high risk of bias were generally smaller (median size 756; range 90–7,925) than those with moderate risk (median size 10,246; range 560–397,852) and low risk (median size 6,992; range 1,448–708,907).

### Meta-Analysis of Unadjusted Data

**Figure 2:**
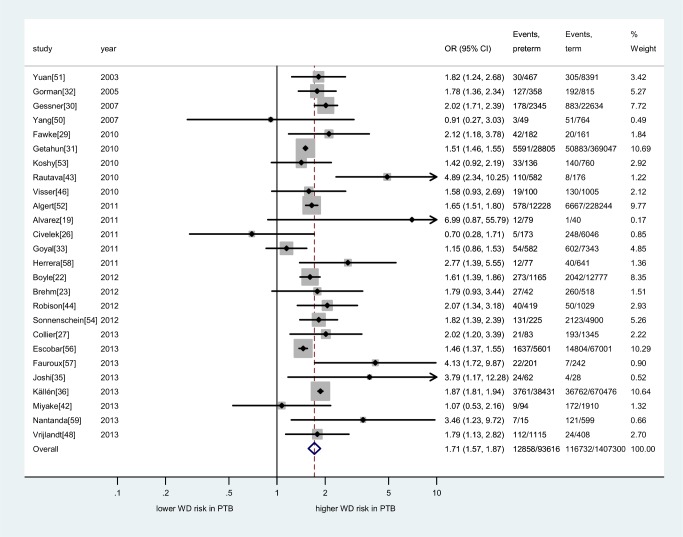
Meta-analysis of unadjusted association between preterm birth and childhood wheezing disorders. Heterogeneity: *I* ^2^ = 82% (95% CI 75%–87%). PTB, preterm birth; WD, wheezing disorders.

Crude data were provided by authors of five studies [26],[33],[46],[53],[54], and were calculated for seven additional studies [19],[30]–[32],[42],[44],[59]. Crude data could not be retrieved for four studies, and these were included in the adjusted meta-analysis only [21],[24],[25],[45]. Pooling of the 26 studies (1,500,916 individuals) for which unadjusted data were available showed a significant association between preterm birth and wheezing disorders (OR 1.71, 95% CI 1.57–1.87, *p*<0.001; Figure 2). Out of 93,616 children born preterm, 12,858 (13.7%) were diagnosed with a wheezing disorder, compared to 116,732 out of 1,407,300 children born at term (8.3%).

### Meta-Analysis of Adjusted Data

**Figure 3:**
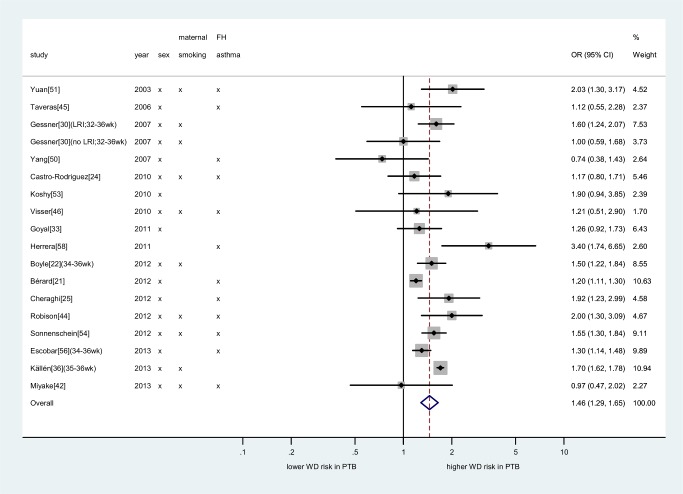
Meta-analysis of adjusted association between preterm birth and childhood wheezing disorders. Subgroups taken from individual studies noted in parentheses. Heterogeneity: *I* ^2^ = 80% (95% CI 68%–86%). Individual study adjustment for the primary confounders is depicted. Additional confounders adjusted for are outlined in Table S3. FH, family history; LRI, lower respiratory infection; PTB, preterm birth; WD, wheezing disorders.

Seventeen studies provided adjusted association measures that could be pooled in a meta-analysis (874,710 individuals). Although the variables for which individual studies adjusted varied (Table S3), the majority included the important confounders: sex, maternal smoking, and parental atopy or asthma. The final summary OR for the association between preterm birth and wheezing disorders was slightly attenuated as compared to the unadjusted analysis (OR 1.46, 95% CI 1.29–1.65, *p*<0.001; Figure 3).

**Figure 4:**
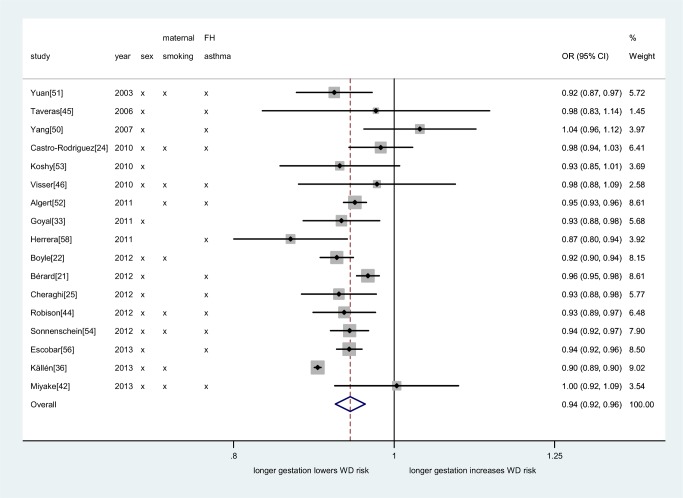
Meta-analysis of adjusted dose–response association between gestational age (per week increase) and childhood wheezing disorders. Heterogeneity: *I* ^2^ = 90% (95% CI 85%–92%). Individual study adjustment for the primary confounders is depicted. Additional confounders adjusted for are outlined in Table S3. FH, family history; WD, wheezing disorders.

In order to investigate a possible “dose–response” relationship between gestational age at birth and wheezing disorder risk, we aggregated corresponding adjusted association measures from 17 studies (1,105,828 individuals; Figure 4). The pooled estimate of the linear association between gestational age and wheezing disorder risk thus obtained (OR 0.94, 95% CI 0.92–0.96, *p*<0.001) indicated a 6% (95% CI 4%–8%) decrease in wheezing disorder risk for every week increase in gestation length up to 40 wk.

### Subgroup Analyses

**Figure 5:**
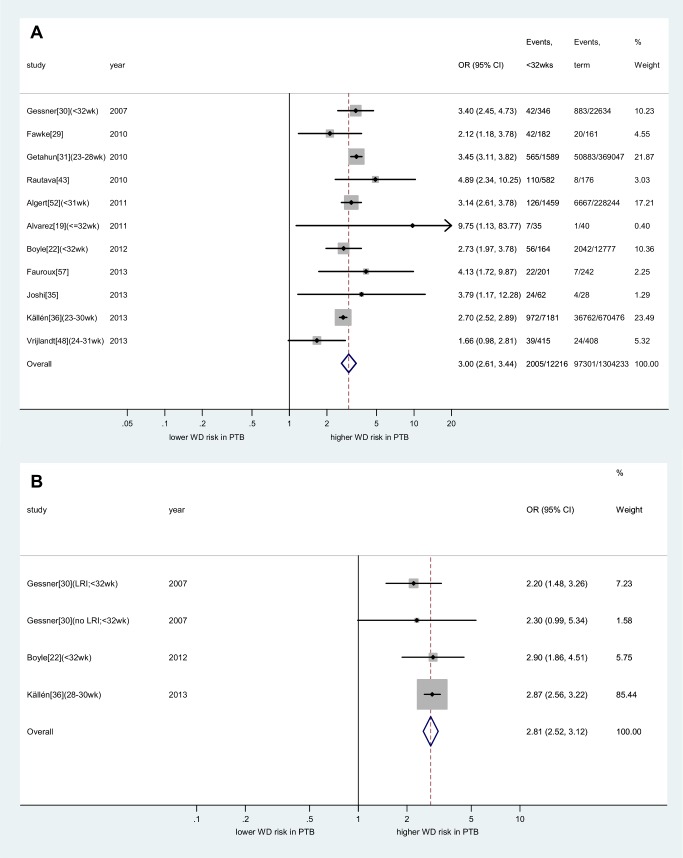
Meta-analysis of association between very preterm birth and childhood wheezing disorders. (A) unadjusted effect estimates; (B) adjusted effect estimates. Subgroups taken from individual studies noted in parentheses. Heterogeneity: *I* ^2^ (unadjusted) = 62% (95% CI 9%–79%); *I* ^2^ (adjusted) = 0% (95% CI 0%–68%). Confounders adjusted for in individual studies are outlined in Figure 3 and Table S3. LRI, lower respiratory infection; PTB, preterm birth; WD, wheezing disorders.

**Figure 6:**
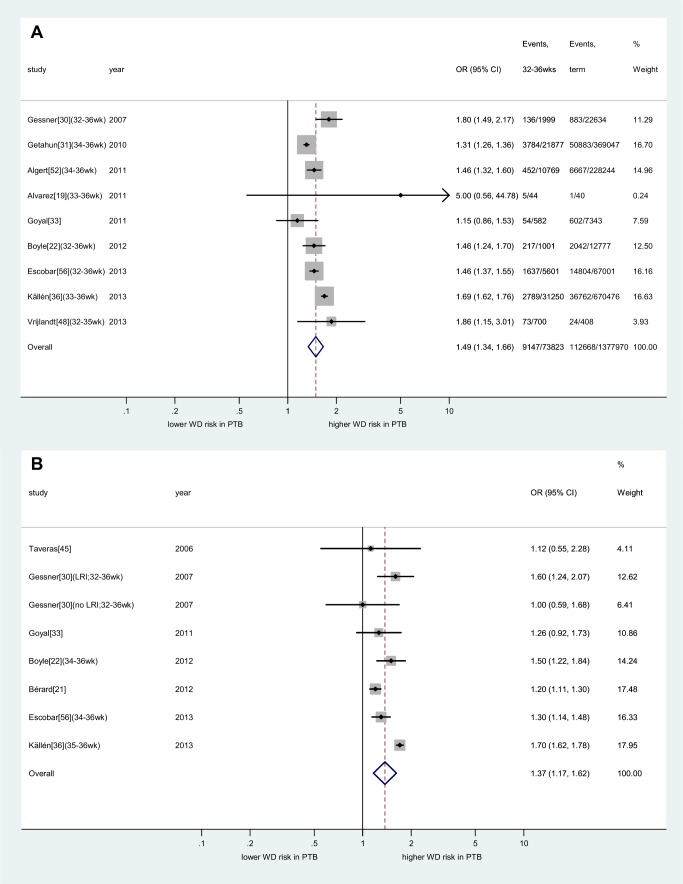
Meta-analysis of association between moderately preterm birth and childhood wheezing disorders. (A) unadjusted effect estimates; (B) adjusted effect estimates. Subgroups taken from individual studies noted in parentheses. Heterogeneity: *I* ^2^ (unadjusted) = 92% (95% CI 87%–94%); *I* ^2^ (adjusted) = 89% (95% CI 82%–93%). Confounders adjusted for in individual studies are outlined in Figure 3 and Table S3. LRI, lower respiratory infection; PTB, preterm birth; WD, wheezing disorders.

The strength of the association between preterm birth and wheezing disorders was similar between children aged <5 y and older children in both unadjusted and adjusted analyses (Figures S1, Figures S2, S3). The risk was considerably higher among children born very preterm (unadjusted: OR 3.00, 95% CI 2.61–3.44, *p*<0.001; adjusted: OR 2.81, 95% CI 2.52–3.12, *p*<0.001; Figure 5) when compared to moderately preterm children (unadjusted: OR 1.49, 95% CI 1.34–1.66, *p*<0.001; adjusted: OR 1.37, 95% CI 1.17–1.62, *p*<0.001; Figure 6).

### Sensitivity Analyses

In adjusted analyses, the association between preterm birth and wheezing disorders was most pronounced in the higher quality studies, whereas studies with a high risk of bias had the highest point estimate in the unadjusted analysis (Figures S4, S5, S6). Aggregate ORs did not differ much between subgroups according to study size (Figures S7, S8, S9), diagnosis ascertainment (Figures S10, S11, S12), or wheezing type (Figures S13, S14, S15).

### Meta-Regression Analysis

To further investigate the independent effects of study size, population age, diagnosis ascertainment, wheezing type, and publication year, meta-regression analysis was performed. None of these factors had an independent effect on the strength of association between preterm birth and wheezing disorders (Table 3). The models' residual *I* ^2^ values suggested that additional unmeasured factors contributed to between-study heterogeneity.

**Table 3:** Meta-regression analysis according to study characteristics.

Factor	Unadjusted Association Measure		Adjusted Association Measure	
	Beta (95% CI)	*p*-Value	Beta (95% CI)	*p*-Value
Study size (per 1,000 individuals)	−0.000 (−0.001 to 0.001)	0.70	0.000 (−0.001 to 0.001)	0.93
Median population age (years)	0.033 (−0.062 to 0.069)	0.92	0.051 (−0.039 to 0.141)	0.24
Asthma (reference = wheezing)	0.102 (−0.250 to 0.453)	0.55	0.035 (−0.348 to 0.417)	0.85
Doctor diagnosis (reference = parental report)	0.009 (−0.333 to 0.352)	0.96	−0.010 (−0.377 to 0.357)	0.95
Year of publication	0.008 (−0.057 to 0.072)	0.80	−0.017 (−0.093 to 0.058)	0.62
Overall		0.99		0.78
Residual *I* ^2^	70%		53%	

### Small-Study Effects

**Figure 7:**
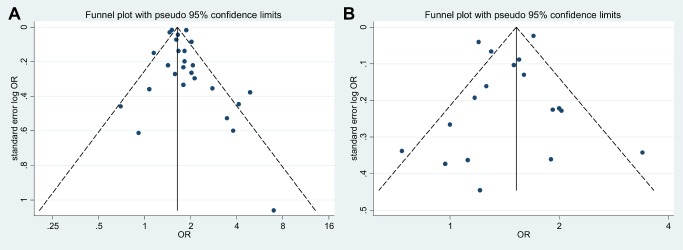
Funnel plots for studies reporting unadjusted and adjusted association measures. (A) unadjusted association measures; (B) adjusted association measures.

Funnel plot asymmetry was minimal for both unadjusted and adjusted association measures (Figure 7). Accordingly, Harbord's test (*p* = 0.55; unadjusted association measures) and Egger's test (*p* = 0.43; adjusted association measures) revealed no important small-study effects potentially indicative of publication bias.

### Population-Attributable Risk

Based on unadjusted association measures, the PAR for childhood wheezing disorders associated with preterm birth was estimated at 4.0% overall, comprising 1.6% (95% CI 1.3%–1.9%) for very preterm birth and 2.4% (95% CI 1.7%–3.1%) for moderately preterm birth. PAR based on adjusted association measures was 3.1%: 1.2% (95% CI 1.1%–1.3%) for very preterm birth and an additional 1.9% (95% CI 1.0%–2.7%) for moderately preterm birth.

## Discussion

In this meta-analysis of observational studies, using data from over 1.5 million children from across six continents, preterm birth was found to be associated with a 1.71 (95% CI 1.57–1.87) times increased risk of childhood wheezing disorders. The association was slightly attenuated by adjustment for potential confounding within individual studies (OR 1.46, 95% CI 1.29–1.65). Additional analyses strongly support a dose–response relationship between length of gestation and wheezing disorders (OR 0.94, 95% CI 0.92–0.96, per week increase), with children born very preterm having three times the risk of that of children born at term (OR 3.00, 95% CI 2.61–3.44). Consistency across sensitivity analyses further supports the robustness of the findings. Moderate preterm birth accounted for the majority of the explained variation in childhood wheezing disorders by preterm birth. Together, these findings are in line with the increasing recognition of the impact that early life influences have on subsequent health and disease, including pulmonary outcomes [1],[8]. Furthermore, they highlight the pressing need for prioritisation of research into prevention of preterm birth and the aetiology of its adverse consequences for subsequent respiratory health.

As with any systematic review we cannot exclude the possibility that potentially relevant studies were missed by our search strategy, although it included the primary online databases for medical research and the most rational search terms relevant to the research question. Identification of additional studies was performed through screening of reference lists and citations, and contacting an international panel of experts. Absence of important small-study effects suggests that bias resulting from selective study inclusion is likely to be minor.

*I* ^2^ values suggested that considerable heterogeneity was present among the studies included in the meta-analysis, although these values may be inflated by the inclusion of some very large studies [60]. Heterogeneity was handled by applying random-effects models [15]. Low *I* ^2^ values in subgroup analyses according to age group suggest that between-study population age differences may contribute. Variation furthermore exists among doctors and parents, and between doctors and parents regarding the definition and perception of wheeze, as well as asthma. We therefore performed sensitivity analyses separating wheezing from asthma, and physician diagnoses from parental reports. The results suggest that, although perceptions may differ, this did not affect the estimation of the association between preterm birth and childhood wheezing disorders. Indeed, residual heterogeneity remained after adjustment for these and other factors via meta-regression analysis. Although study quality may be another responsible factor, the observation that aggregated association measures were most pronounced in studies with low risk of bias supports the validity of the conclusions.

Several factors potentially confound the association between preterm birth and wheezing disorders [9]. We therefore performed separate analyses aggregating adjusted association measures. This attenuated the association between preterm birth and wheezing disorders to some degree, as expected. The vast majority of studies adjusted for important potential confounders, although their number and nature varied between studies. A varying degree of residual confounding thus remains, which should be taken into account when interpreting the findings. From studies reporting association measures according to multiple gestational age strata we included the least preterm stratum. The aggregated point estimate is therefore likely to underestimate the actual association.

Different methods for gestational age estimation exist. Although most studies failed to report their approach, first trimester ultrasound is increasingly used in higher income countries. Alternative estimation is usually based on the last menstrual period, which is known to overestimate the numbers of preterm and post-term births [61]. Again, this is expected to result in underestimation of the association between preterm birth and wheezing disorders [62].

In a previous meta-analysis, preterm birth was associated with a 1.37 (95% CI 1.30–1.43) times increased risk of asthma [9]. To better reflect current neonatal practice, we included only cohorts born from the 1990s onwards, resulting in a much larger sample size and negligible overlap with the report by Jaakkola and colleagues [9]. The substantially greater numbers of events in our review enabled us—for the first time—to investigate aggregated adjusted association estimates to account for potential confounding, explore the association with degree of prematurity (i.e., a dose-dependent relationship), and estimate the associated disease burden (i.e., PAR). These additional steps are crucial to interpreting these data, as the evidence is of necessity derived from observational studies, which are inherently at risk of bias. We can only speculate as to the underlying reasons for the association being more pronounced in our report, even after adjustment for confounding. The former review mainly included children born in the 1960s–1980s [9]. These older cohorts are likely to be less preterm on average, which may explain their somewhat lower risk of developing asthma. Analysis by study characteristics in both meta-analyses suggests that the study characteristics are unlikely to explain the difference. The apparent increase over time in wheezing disorder risk associated with preterm birth indicates that changes in neonatal practice have generally failed to improve obstructive pulmonary outcomes in children born preterm.

The current findings do not support prior suggestions that the association between preterm birth and wheezing disorders becomes less prominent with increasing age [9]. Instead, the strength of the association was similar across age groups, suggesting that the pulmonary consequences of preterm birth tend to persist throughout the life course. This observation is supported by evidence from longitudinal studies showing temporal tracking of small airway disease among individuals born preterm [54],[56],[63], and by recent confirmation of a link between preterm birth and chronic obstructive pulmonary disease [8]. The contribution of atopy to this association is unclear and requires further study.

The processes underlying preterm birth are poorly understood [64]. The incidence as well as the underlying causes of preterm birth are known to vary both geographically and temporally, at least partially driven by meteorological, socioeconomic, ethnic, and (epi)genetic variation [2],[64]–[66]. Environmental exposures, such as pollution and tobacco smoke, and individual behavioural differences (i.e., hygiene, smoking during pregnancy) furthermore contribute [64],[65]. Many such factors have also been linked to the development of wheezing disorders [40],[44],[67]. Through interference with the foetus's natural environment during critical windows of development, the processes underlying preterm birth challenge the developmental plasticity of the lungs and airways [1],[4],[43]. Common antecedents of preterm birth such as inflammation, maternal smoking, metabolic derangement, hypoxia, and growth restriction have well-recognised adverse effects on lung maturation and structure, T cell polarisation and development, and airway reactivity [4],[31],[67]–[69]. Such alterations can differentially affect susceptibility of the lungs to injurious exposures that commonly follow preterm birth, including sepsis, respiratory infections, mechanical ventilation, and hyperoxia [11],[70]–[72]. For any of these factors, the likelihood of being exposed increases with decreasing gestational age at birth. Immediate adverse effects are seen in very preterm infants developing BPD, which is an independent risk factor for reactive airway disease [5],[29],[35],[49],[67]. Preterm birth furthermore augments the association of several of these risk factors, such as antenatal inflammation and smoke exposure, with childhood wheezing [31],[44]. Such mechanisms may all contribute to the observed dose–response relationship between gestational age and adverse respiratory outcomes, including wheezing disorders [4],[31]. Genetic influences and gene–environment interactions are furthermore likely to play a role, and the link between preterm birth and asthma has been suggested to at least partially reflect a common genetic background [9],[73].

The magnitude of the problem having been established, there is now a pressing need to address several knowledge gaps. Epidemiological studies should concentrate on temporal tracking of respiratory status into adulthood following preterm birth, with particular focus on different wheezing phenotypes, and estimating the relative impact of distinct early life factors on these patterns. Recent accomplishments in combining European birth cohorts can help provide the required numbers [74]. There is, furthermore, a need for additional studies from low- and middle-income countries. More high-quality research is required to identify the underlying mechanisms and accordingly develop appropriate preventive and therapeutic measures. Innovative approaches may combine these aspects, as recently highlighted by a randomised controlled trial of repeated administration of a monoclonal respiratory syncytial virus antibody in moderately preterm infants [75]. The intervention induced an important decrease in wheezing in the first year, establishing the causal link between respiratory syncytial virus and wheezing, as well as offering a possible solution [75]. Immune modulation is beginning to fulfil its promise in neonatal medicine, and preterm babies may well prove particularly favourable targets for the immunomodulatory therapies that are likely to shape the future of asthma management [69],[76],[77]. The potential for stratified medicine to benefit children born preterm needs to be more generally explored through appropriately designed trials and pre-specified subgroup analyses in future trials.

This work provides compelling evidence that preterm birth is an important early life risk factor for wheezing disorders in childhood. Given the increasing incidence of both entities and their potentially lifelong consequences, there is an urgent need to identify the underlying mechanisms and explore the potential for preventive and therapeutic approaches.

## Supporting Information

Checklist S1 **PRISMA checklist.**
(DOC)

Figure S1 **Meta-analysis of unadjusted association between preterm birth and childhood wheezing disorders according to age group.** Since estimates from Boyle [22] and Vrijlandt [48] are included on both sides of the comparison, overall association measures are not displayed. Subgroups taken from individual studies noted in parentheses. Heterogeneity: *I* ^2^ (<5 y) = 56% (95% CI 0%–76%); *I* ^2^ (≥5 y) = 48% (95% CI 0%–72%). PTB, preterm birth; WD, wheezing disorders.
(TIF)

Figure S2 **Meta-analysis of adjusted association between preterm birth and childhood wheezing disorders according to age group.** Since estimates from Boyle [22] are included on both sides of the comparison, overall association measures are not displayed. Subgroups taken from individual studies noted in parentheses. Confounders adjusted for in individual studies are outlined in Figure 3 and Table S3. Heterogeneity: *I* ^2^ (<5 y) = 31% (95% CI 0%–77%); *I* ^2^ (≥5 y) = 41% (95% CI 0%–69%). PTB, preterm birth; WD, wheezing disorders.
(TIF)

Figure S3 **Meta-analysis of adjusted dose–response association between gestational age (per week increase) and childhood wheezing disorders according to age group.** Since estimates from Boyle [22] are included on both sides of the comparison, overall association measures are not displayed. Subgroups taken from individual studies noted in parentheses. Confounders adjusted for in individual studies are outlined in Figure 4 and Table S3. Heterogeneity: *I* ^2^ (<5 y) = 0% (95% CI 0%–68%); *I* ^2^ (≥5 y) = 37% (95% CI 0%–69%). WD, wheezing disorders.
(TIF)

Figure S4 **Meta-analysis of unadjusted association between preterm birth and childhood wheezing disorders according to risk of bias.** Heterogeneity: *I* ^2^ (high risk of bias) = 60% (95% CI 10%–77%); *I* ^2^ (moderate risk of bias) = 63% (95% CI 0%–80%); *I* ^2^ (low risk of bias) = 0% (95% CI 0%–68%). PTB, preterm birth; WD, wheezing disorders.
(TIF)

Figure S5 **Meta-analysis of adjusted association between preterm birth and childhood wheezing disorders according to risk of bias.** Subgroups taken from individual studies noted in parentheses. Confounders adjusted for in individual studies are outlined in Figure 3 and Table S3. Heterogeneity: *I* ^2^ (high risk of bias) = 56% (95% CI 0%–82%); *I* ^2^ (moderate risk of bias) = 46% (95% CI 0%–73%); *I* ^2^ (low risk of bias) = 0% (95% CI 0%–68%). PTB, preterm birth; WD, wheezing disorders.
(TIF)

Figure S6 **Meta-analysis of adjusted dose–response association between gestational age (per week increase) and childhood wheezing disorders according to risk of bias.** Confounders adjusted for in individual studies are outlined in Figure 4 and Table S3. Heterogeneity: *I* ^2^ (high risk of bias) = 39% (95% CI 0%–76%); *I* ^2^ (moderate risk of bias) = 63% (95% CI 0%–81%); *I* ^2^ (low risk of bias) = 8% (95% CI 2%–90%). WD, wheezing disorders.
(TIF)

Figure S7 **Meta-analysis of unadjusted association between preterm birth and childhood wheezing disorders according to study size.** Heterogeneity: *I* ^2^ (*n*<10,000) = 47% (95% CI 0%–67%); *I* ^2^ (*n*≥10,000) = 95% (95% CI 93%–97%). PTB, preterm birth; WD, wheezing disorders.
(TIF)

Figure S8 **Meta-analysis of adjusted association between preterm birth and childhood wheezing disorders according to study size.** Subgroups taken from individual studies noted in parentheses. Confounders adjusted for in individual studies are outlined in Figure 3 and Table S3. Heterogeneity: *I* ^2^ (*n*<10,000) = 42% (95% CI 0%–68%); *I* ^2^ (*n*≥10,000) = 94% (95% CI 89%–96%). PTB, preterm birth; WD, wheezing disorders.
(TIF)

Figure S9 **Meta-analysis of adjusted dose–response association between gestational age (per week increase) and childhood wheezing disorders according to study size.** Confounders adjusted for in individual studies are outlined in Figure 4 and Table S3. Heterogeneity: *I* ^2^ (*n*<10,000) = 39% (95% CI 0%–68%); *I* ^2^ (*n*≥10,000) = 97% (95% CI 95%–98%). WD, wheezing disorders.
(TIF)

Figure S10 **Meta-analysis of unadjusted association between preterm birth and childhood wheezing disorders according to diagnosis ascertainment.** Heterogeneity: *I* ^2^ (parent reported) = 19% (95% CI 0%–55%); *I* ^2^ (doctor diagnosed) = 93% (95% CI 89%–95%). PTB, preterm birth; WD, wheezing disorders.
(TIF)

Figure S11 **Meta-analysis of adjusted association between preterm birth and childhood wheezing disorders according to diagnosis ascertainment.** Subgroups taken from individual studies noted in parentheses. Confounders adjusted for in individual studies are outlined in Figure 3 and Table S3. Heterogeneity: *I* ^2^ (parent reported) = 43% (95% CI 0%–71%); *I* ^2^ (doctor diagnosed) = 90% (95% CI 82%–93%). PTB, preterm birth; WD, wheezing disorders.
(TIF)

Figure S12 **Meta-analysis of adjusted dose–response association between gestational age (per week increase) and childhood wheezing disorders according to diagnosis ascertainment.** Confounders adjusted for in individual studies are outlined in Figure 4 and Table S3. Heterogeneity: *I* ^2^ (parent reported) = 54% (95% CI 0%–76%); *I* ^2^ (doctor diagnosed) = 95% (95% CI 92%–96%). WD, wheezing disorders.
(TIF)

Figure S13 **Meta-analysis of unadjusted association between preterm birth and childhood wheezing disorders according to wheezing type.** Heterogeneity: *I* ^2^ (wheezing) = 52% (95% CI 0%–76%); *I* ^2^ (asthma) = 86% (95% CI 79%–90%). PTB, preterm birth; WD, wheezing disorders.
(TIF)

Figure S14 **Meta-analysis of adjusted association between preterm birth and childhood wheezing disorders according to wheezing type.** Subgroups taken from individual studies noted in parentheses. Confounders adjusted for in individual studies are outlined in Figure 3 and Table S3. Heterogeneity: *I* ^2^ (wheezing) = 41% (95% CI 0%–74%); *I* ^2^ (asthma) = 86% (95% CI 76%–90%). PTB, preterm birth; WD, wheezing disorders.
(TIF)

Figure S15 **Meta-analysis of adjusted dose–response association between gestational age (per week increase) and childhood wheezing disorders according to wheezing type.** Confounders adjusted for in individual studies are outlined in Figure 4 and Table S3. Heterogeneity: *I* ^2^ (wheezing) = 52% (95% CI 0%–78%); *I* ^2^ (asthma) = 92% (95% CI 87%–94%). WD, wheezing disorders.
(TIF)

Table S1 **Characteristics of studies excluded because of population overlap.** *Selected inhaled or systemic adrenergics, anticholinergics, xantines, antiallergics, leukotriene receptor antagonists, and/or steroids; ** as compared to preterm babies without BPD. GA, gestational age; KFHP, Kaiser Foundation Health Plan; KPMCP, Kaiser Permanente Medical Care Program; N/R, not reported; LMP, last menstrual period; SD, standard deviation.
(DOCX)

Table S2 **Assessment of study quality.** Scoring according to the Effective Public Health Practice Project quality assessment tool for quantitative studies. For study design “7” indicates cross-sectional design in all cases. N/A, not applicable.
(DOCX)

Table S3 **Covariates in multivariate models of individual studies associating preterm birth with wheezing disorders.** ^a^month of conception, maternal urinary tract infection, pre-labour rupture of membranes, prostaglandin induction, neonatal jaundice, neonatal sepsis; ^b^threatened miscarriage, infections, asphyxia, birth trauma, premature rupture of membranes, umbilical cord knots, placenta problems; ^c^CC10 G+38A polymorphisms, cord blood IgE.
(DOCX)

Text S1 **PROSPERO-registered review protocol.**
(PDF)

Text S2 **Expert panel.**
(DOCX)

## Abbreviations

BPD: bronchopulmonary dysplasia
OR: odds ratio
PAR: population-attributable risk
