# Supplementary figures and images for: Preterm Birth and Childhood Wheezing Disorders: A Systematic Review and Meta-Analysis
Source: PLoS Med. 2014 Jan 28;11(1):e1001596. doi: 10.1371/journal.pmed.1001596 (PMC3904844; doi:10.1371/journal.pmed.1001596)

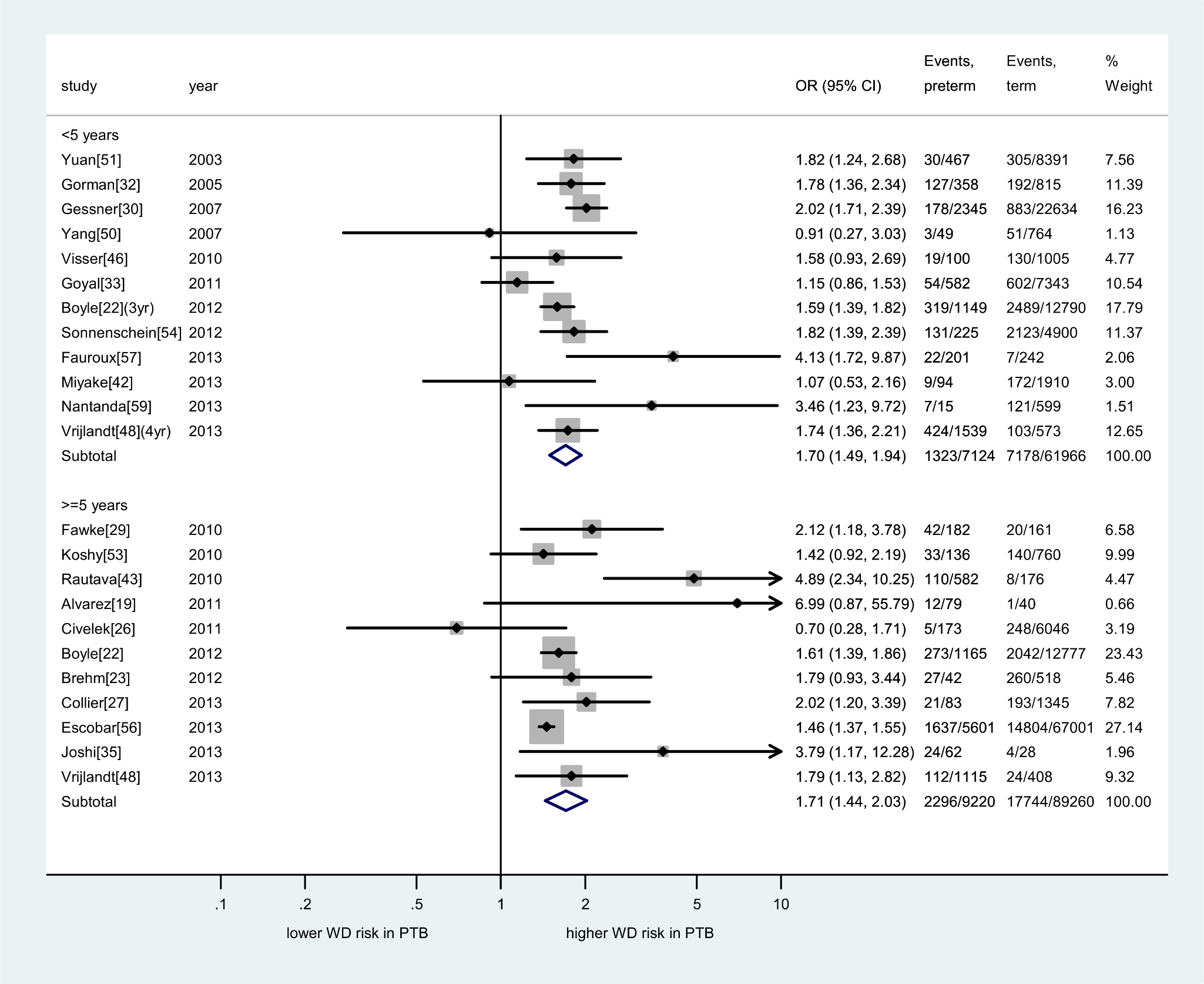

Supplement: Figure S1 — Meta-analysis of unadjusted association between preterm birth and childhood wheezing disorders according to age group. Since estimates from Boyle [22] and Vrijlandt [48] are included on both sides of the comparison, overall association measures are not displayed. Subgroups taken from individual studies noted in parentheses. Heterogeneity: I 2 (<5 y) = 56% (95% CI 0%–76%); I 2 (≥5 y) = 48% (95% CI 0%–72%). PTB, preterm birth; WD, wheezing disorders. (TIF) [file pmed.1001596.s002.tif]

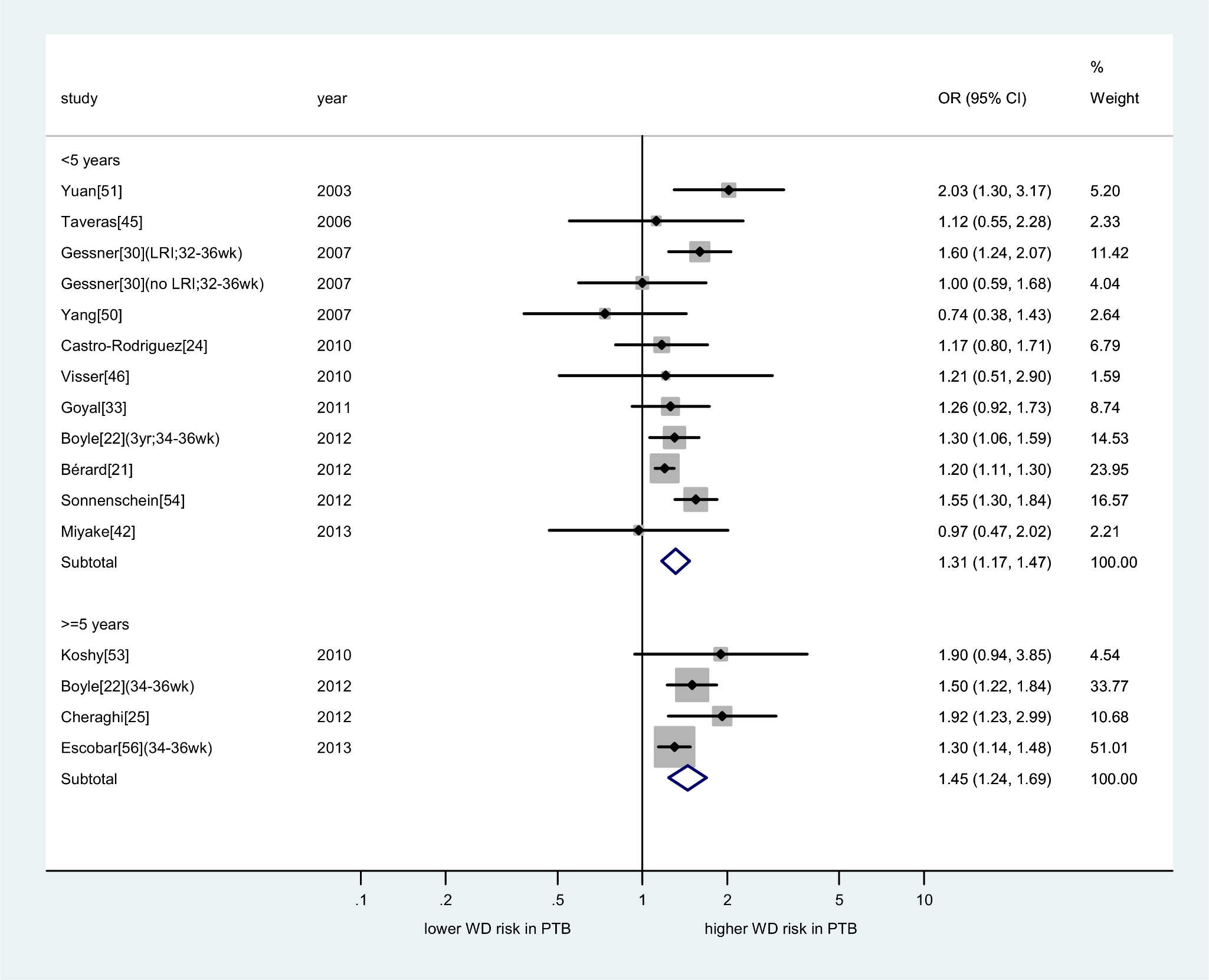

Supplement: Figure S2 — Meta-analysis of adjusted association between preterm birth and childhood wheezing disorders according to age group. Since estimates from Boyle [22] are included on both sides of the comparison, overall association measures are not displayed. Subgroups taken from individual studies noted in parentheses. Confounders adjusted for in individual studies are outlined in Figure 3 and Table S3. Heterogeneity: I 2 (<5 y) = 31% (95% CI 0%–77%); I 2 (≥5 y) = 41% (95% CI 0%–69%). PTB, preterm birth; WD, wheezing disorders. (TIF) [file pmed.1001596.s003.tif]

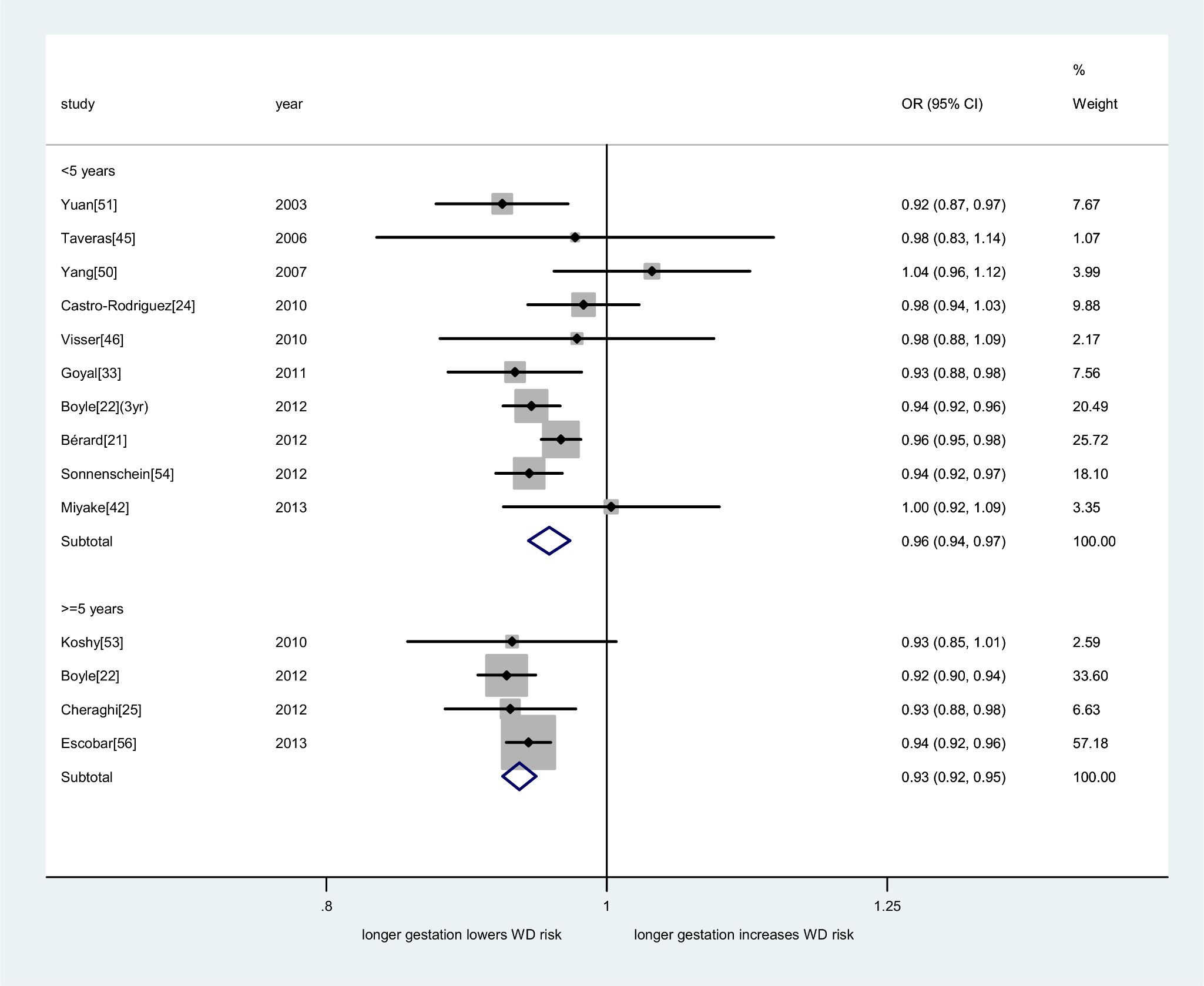

Supplement: Figure S3 — Meta-analysis of adjusted dose–response association between gestational age (per week increase) and childhood wheezing disorders according to age group. Since estimates from Boyle [22] are included on both sides of the comparison, overall association measures are not displayed. Subgroups taken from individual studies noted in parentheses. Confounders adjusted for in individual studies are outlined in Figure 4 and Table S3. Heterogeneity: I 2 (<5 y) = 0% (95% CI 0%–68%); I 2 (≥5 y) = 37% (95% CI 0%–69%). WD, wheezing disorders. (TIF) [file pmed.1001596.s004.tif]

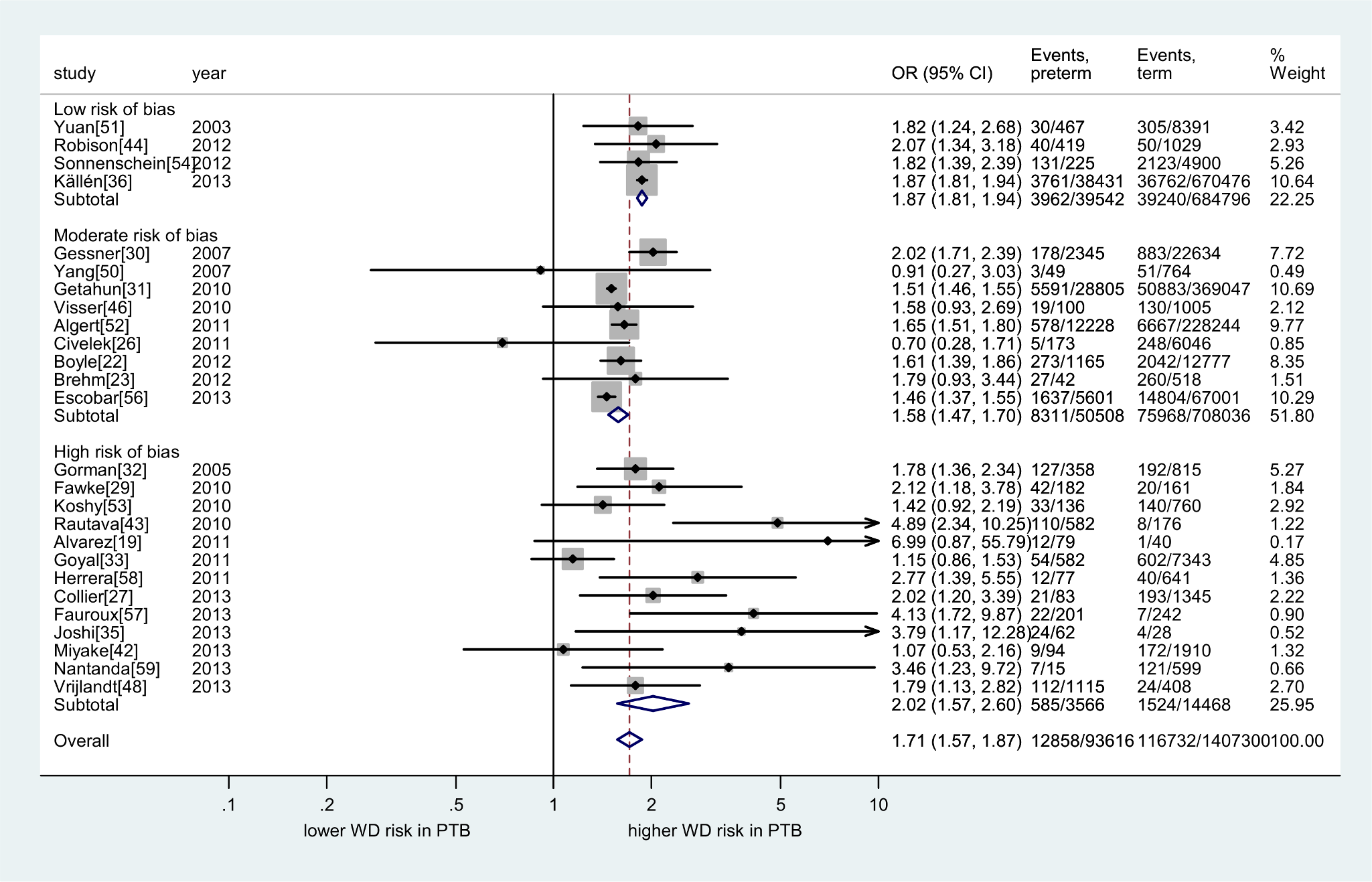

Supplement: Figure S4 — Meta-analysis of unadjusted association between preterm birth and childhood wheezing disorders according to risk of bias. Heterogeneity: I 2 (high risk of bias) = 60% (95% CI 10%–77%); I 2 (moderate risk of bias) = 63% (95% CI 0%–80%); I 2 (low risk of bias) = 0% (95% CI 0%–68%). PTB, preterm birth; WD, wheezing disorders. (TIF) [file pmed.1001596.s005.tif]

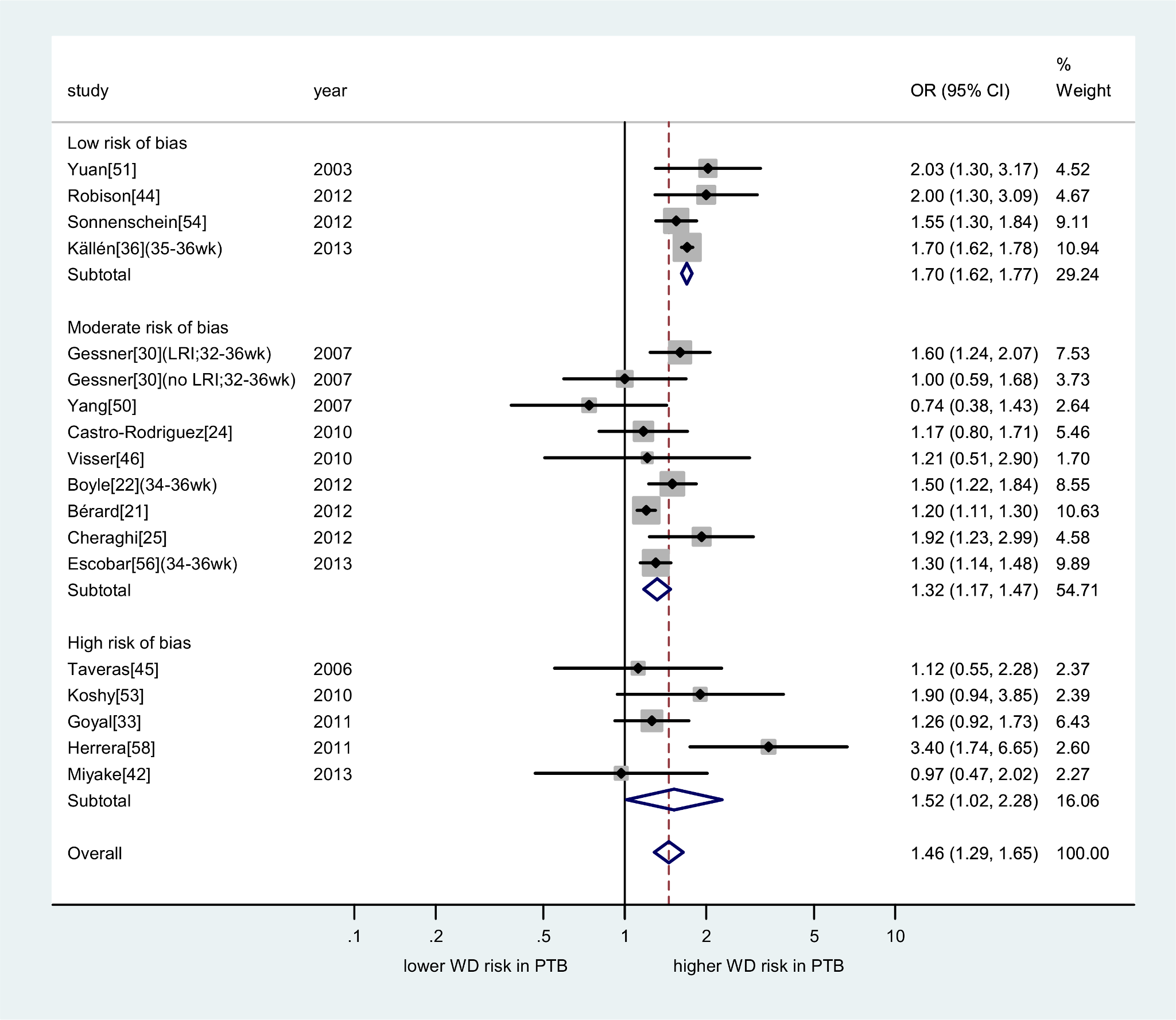

Supplement: Figure S5 — Meta-analysis of adjusted association between preterm birth and childhood wheezing disorders according to risk of bias. Subgroups taken from individual studies noted in parentheses. Confounders adjusted for in individual studies are outlined in Figure 3 and Table S3. Heterogeneity: I 2 (high risk of bias) = 56% (95% CI 0%–82%); I 2 (moderate risk of bias) = 46% (95% CI 0%–73%); I 2 (low risk of bias) = 0% (95% CI 0%–68%). PTB, preterm birth; WD, wheezing disorders. (TIF) [file pmed.1001596.s006.tif]

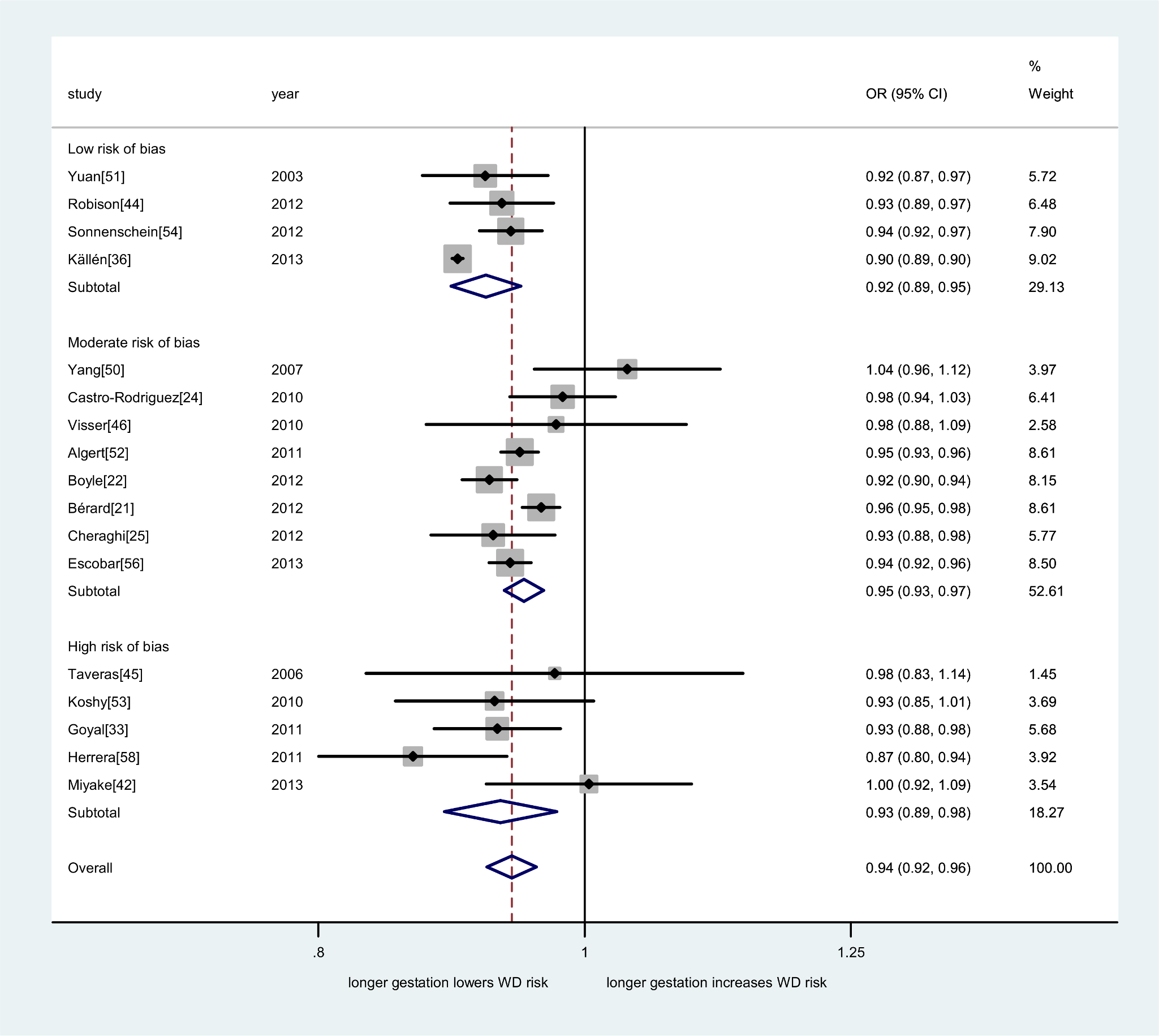

Supplement: Figure S6 — Meta-analysis of adjusted dose–response association between gestational age (per week increase) and childhood wheezing disorders according to risk of bias. Confounders adjusted for in individual studies are outlined in Figure 4 and Table S3. Heterogeneity: I 2 (high risk of bias) = 39% (95% CI 0%–76%); I 2 (moderate risk of bias) = 63% (95% CI 0%–81%); I 2 (low risk of bias) = 8% (95% CI 2%–90%). WD, wheezing disorders. (TIF) [file pmed.1001596.s007.tif]

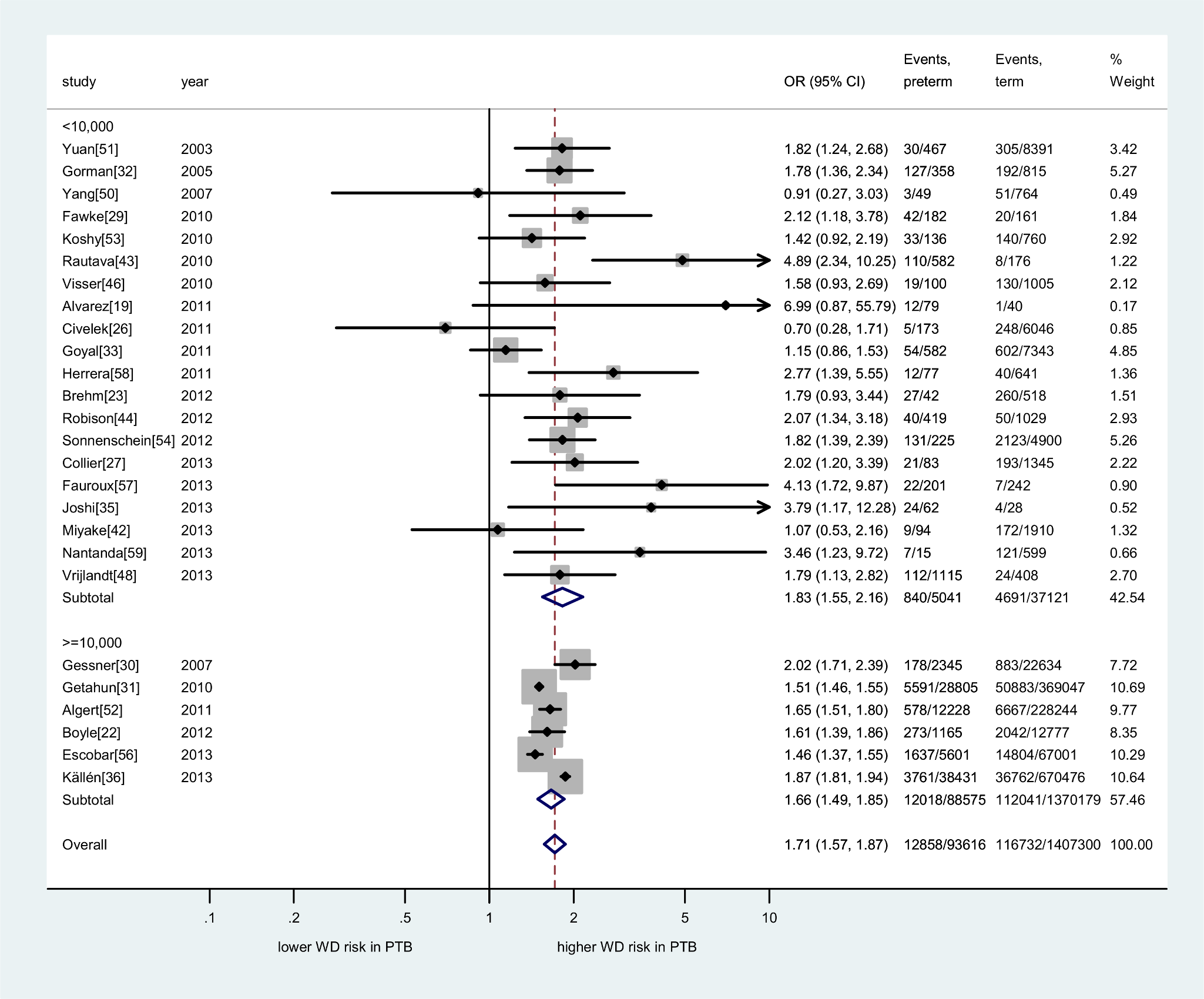

Supplement: Figure S7 — Meta-analysis of unadjusted association between preterm birth and childhood wheezing disorders according to study size. Heterogeneity: I 2 (n<10,000) = 47% (95% CI 0%–67%); I 2 (n≥10,000) = 95% (95% CI 93%–97%). PTB, preterm birth; WD, wheezing disorders. (TIF) [file pmed.1001596.s008.tif]

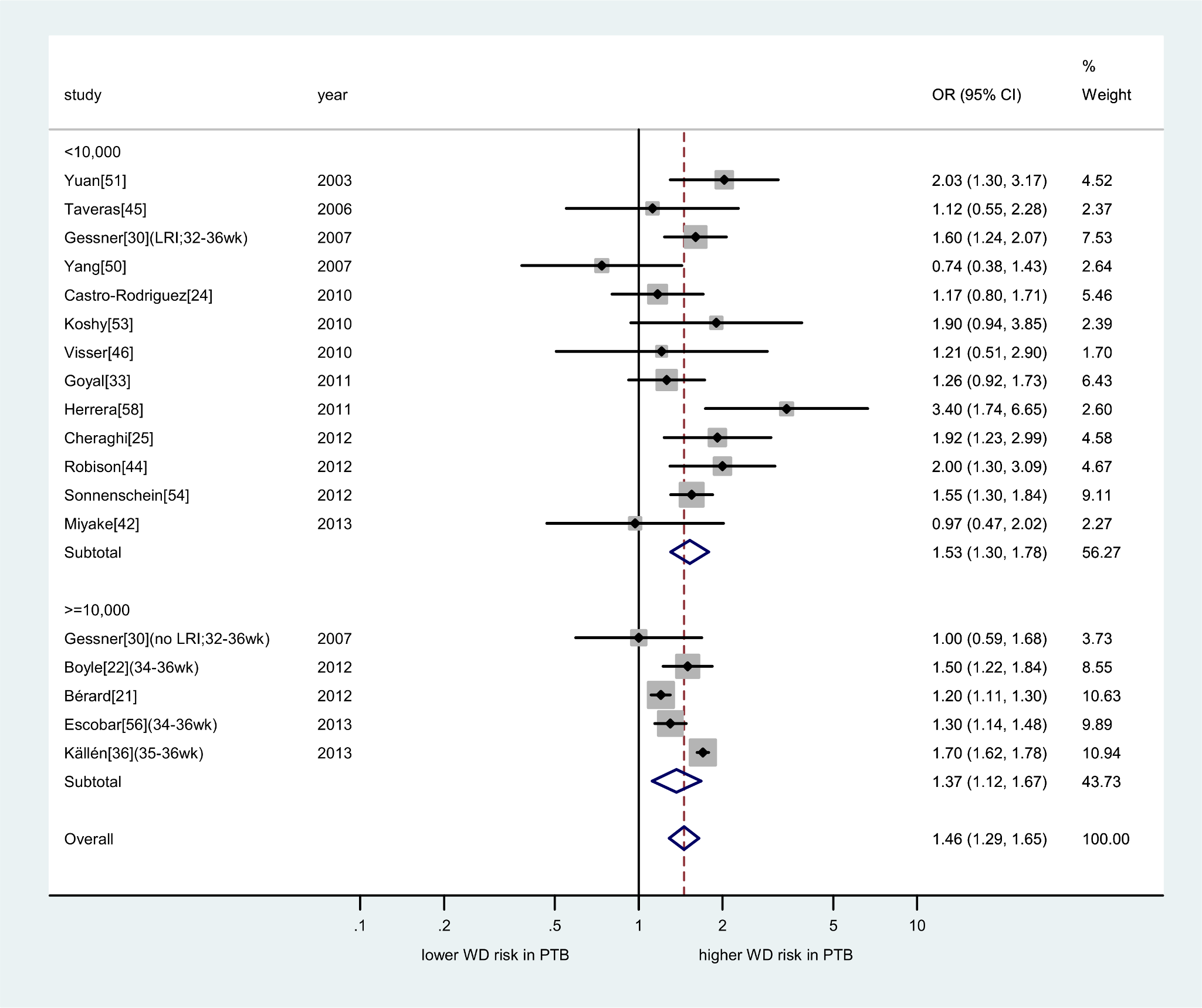

Supplement: Figure S8 — Meta-analysis of adjusted association between preterm birth and childhood wheezing disorders according to study size. Subgroups taken from individual studies noted in parentheses. Confounders adjusted for in individual studies are outlined in Figure 3 and Table S3. Heterogeneity: I 2 (n<10,000) = 42% (95% CI 0%–68%); I 2 (n≥10,000) = 94% (95% CI 89%–96%). PTB, preterm birth; WD, wheezing disorders. (TIF) [file pmed.1001596.s009.tif]

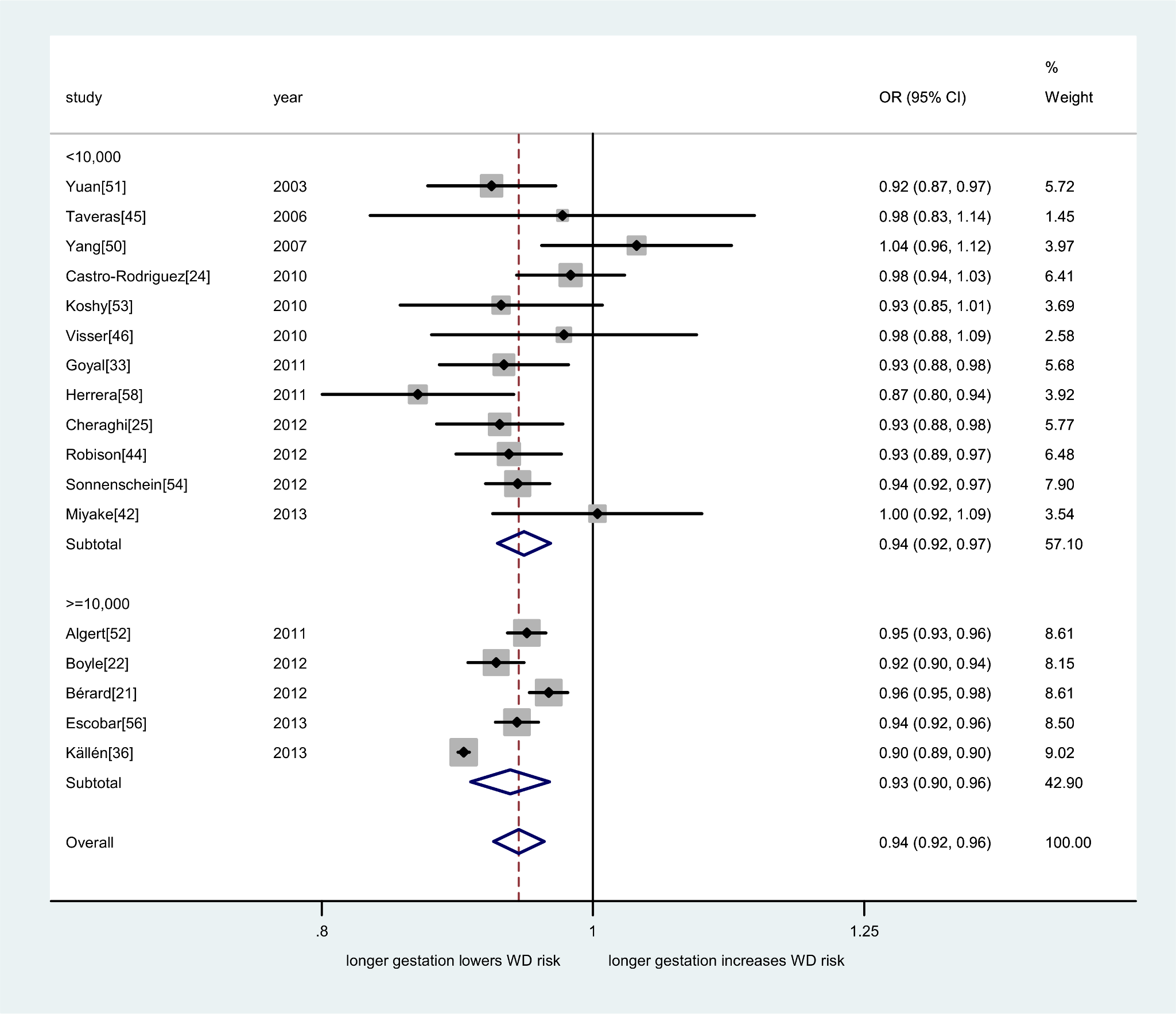

Supplement: Figure S9 — Meta-analysis of adjusted dose–response association between gestational age (per week increase) and childhood wheezing disorders according to study size. Confounders adjusted for in individual studies are outlined in Figure 4 and Table S3. Heterogeneity: I 2 (n<10,000) = 39% (95% CI 0%–68%); I 2 (n≥10,000) = 97% (95% CI 95%–98%). WD, wheezing disorders. (TIF) [file pmed.1001596.s010.tif]

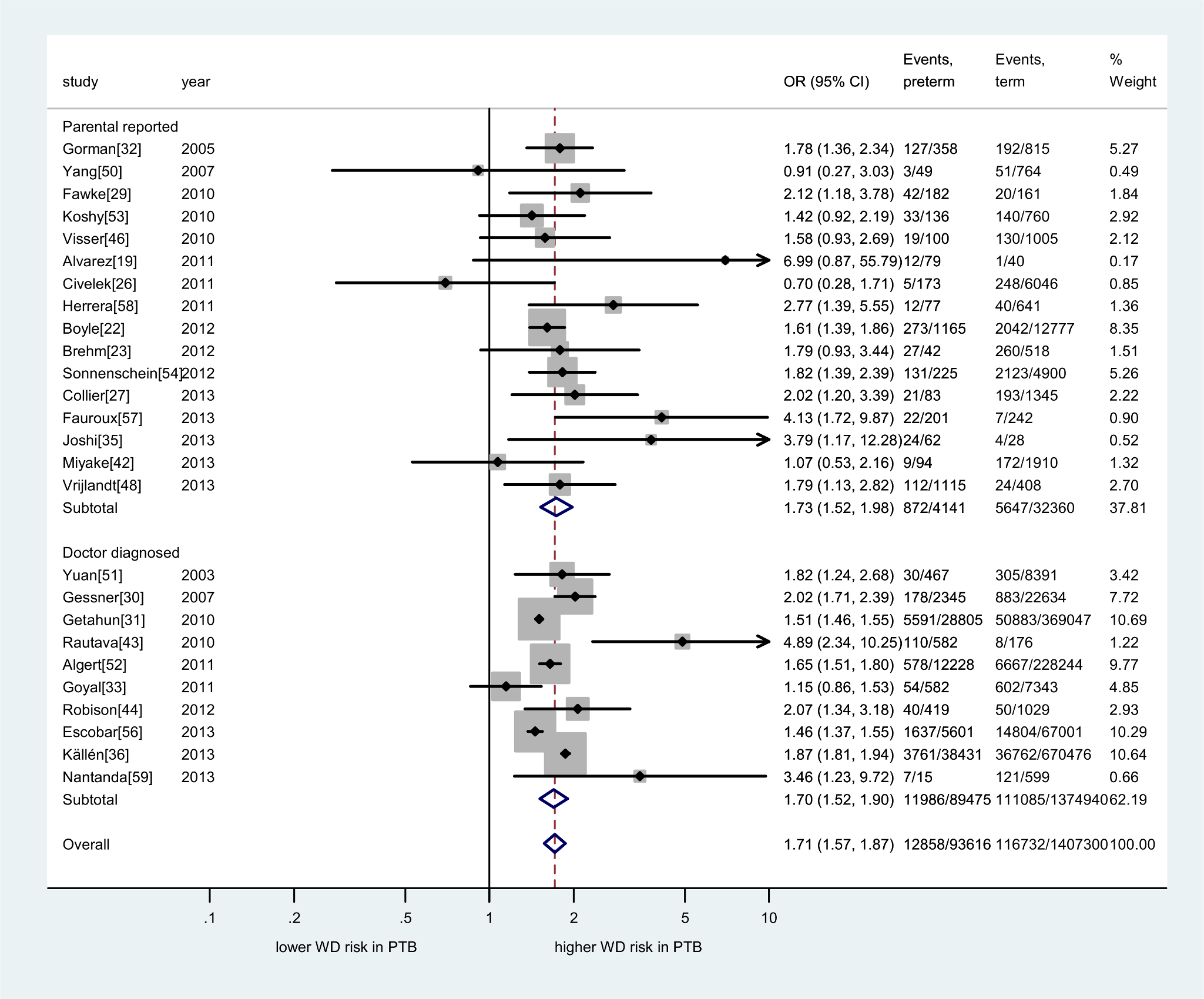

Supplement: Figure S10 — Meta-analysis of unadjusted association between preterm birth and childhood wheezing disorders according to diagnosis ascertainment. Heterogeneity: I 2 (parent reported) = 19% (95% CI 0%–55%); I 2 (doctor diagnosed) = 93% (95% CI 89%–95%). PTB, preterm birth; WD, wheezing disorders. (TIF) [file pmed.1001596.s011.tif]

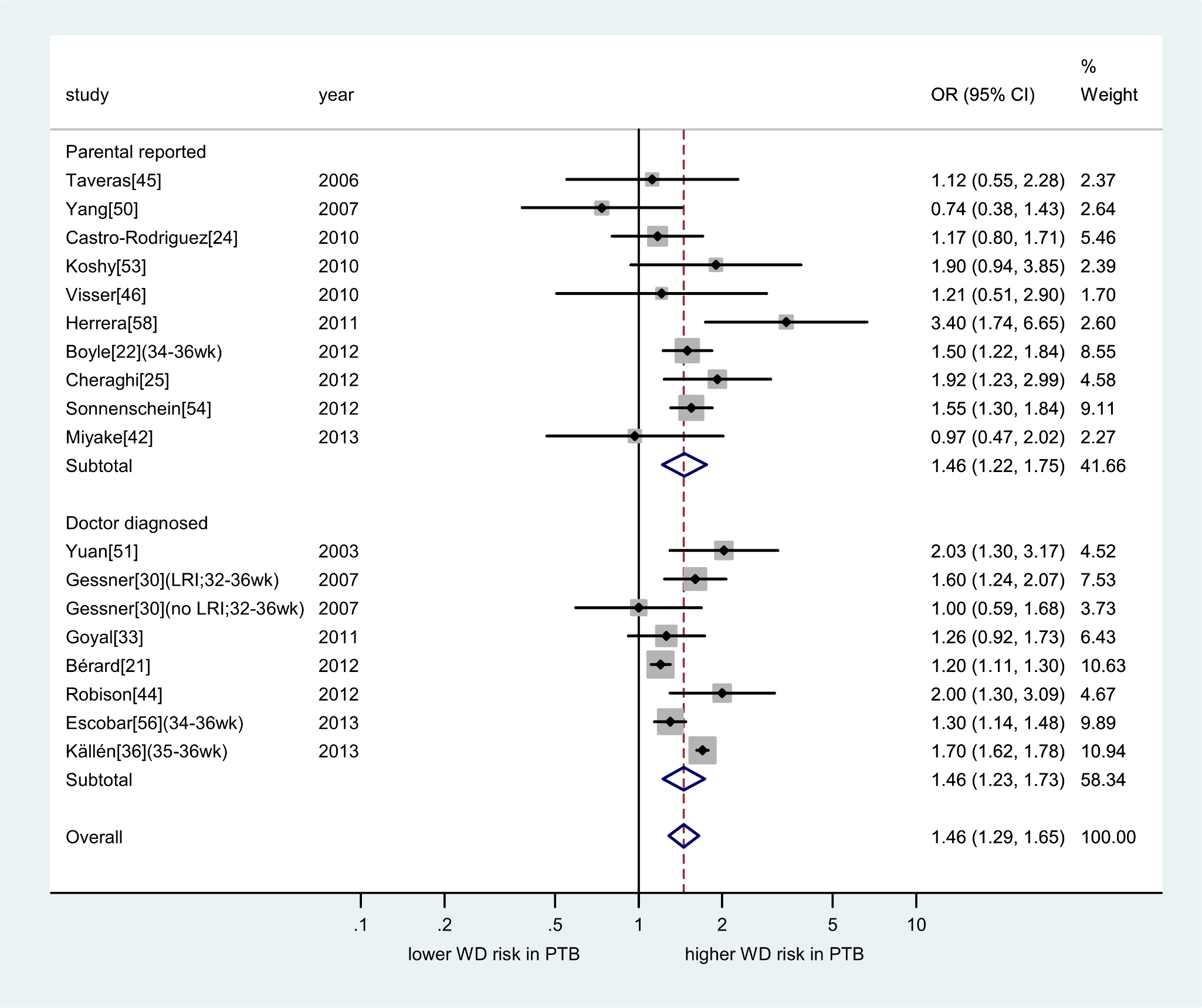

Supplement: Figure S11 — Meta-analysis of adjusted association between preterm birth and childhood wheezing disorders according to diagnosis ascertainment. Subgroups taken from individual studies noted in parentheses. Confounders adjusted for in individual studies are outlined in Figure 3 and Table S3. Heterogeneity: I 2 (parent reported) = 43% (95% CI 0%–71%); I 2 (doctor diagnosed) = 90% (95% CI 82%–93%). PTB, preterm birth; WD, wheezing disorders. (TIF) [file pmed.1001596.s012.tif]

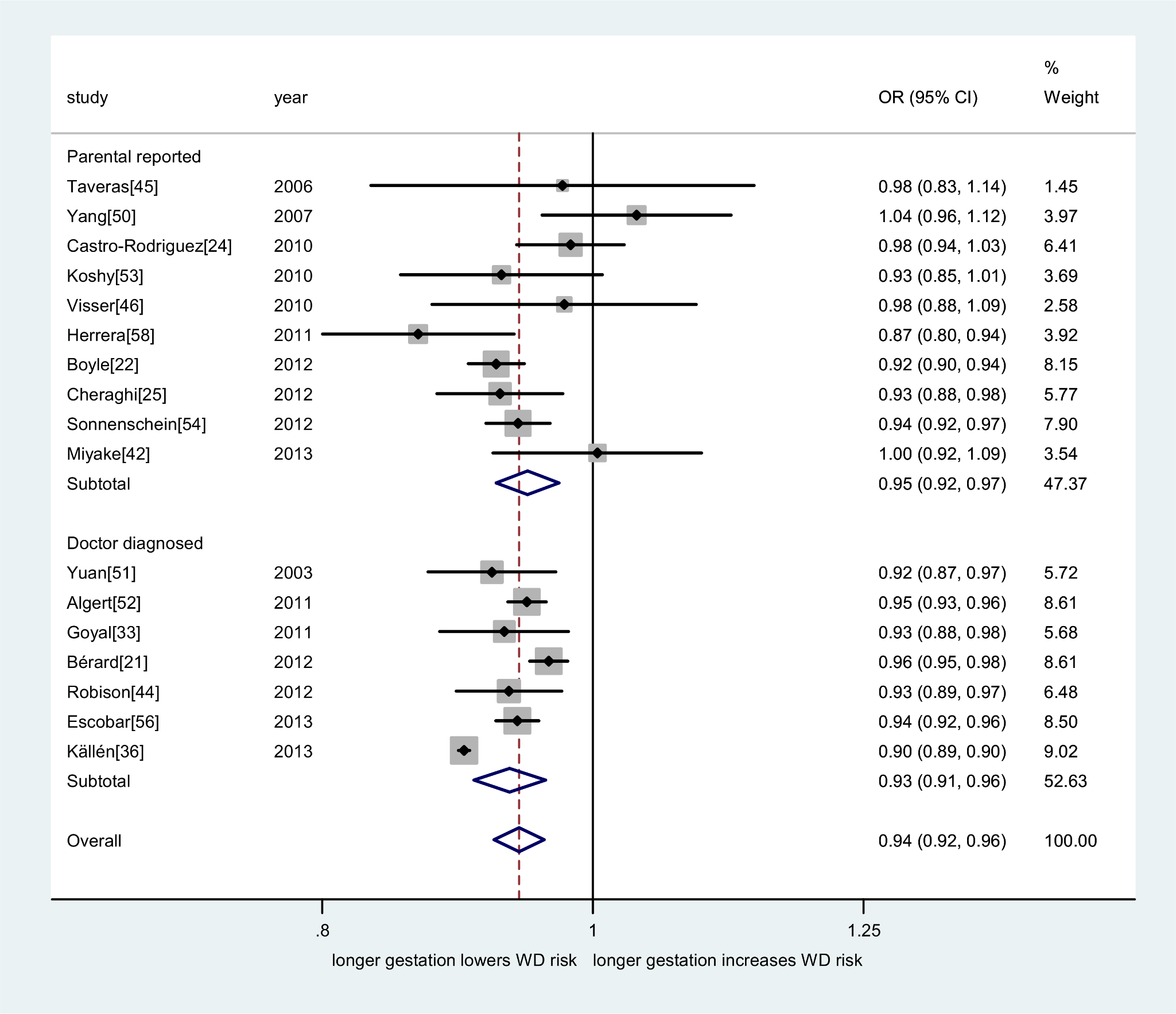

Supplement: Figure S12 — Meta-analysis of adjusted dose–response association between gestational age (per week increase) and childhood wheezing disorders according to diagnosis ascertainment. Confounders adjusted for in individual studies are outlined in Figure 4 and Table S3. Heterogeneity: I 2 (parent reported) = 54% (95% CI 0%–76%); I 2 (doctor diagnosed) = 95% (95% CI 92%–96%). WD, wheezing disorders. (TIF) [file pmed.1001596.s013.tif]

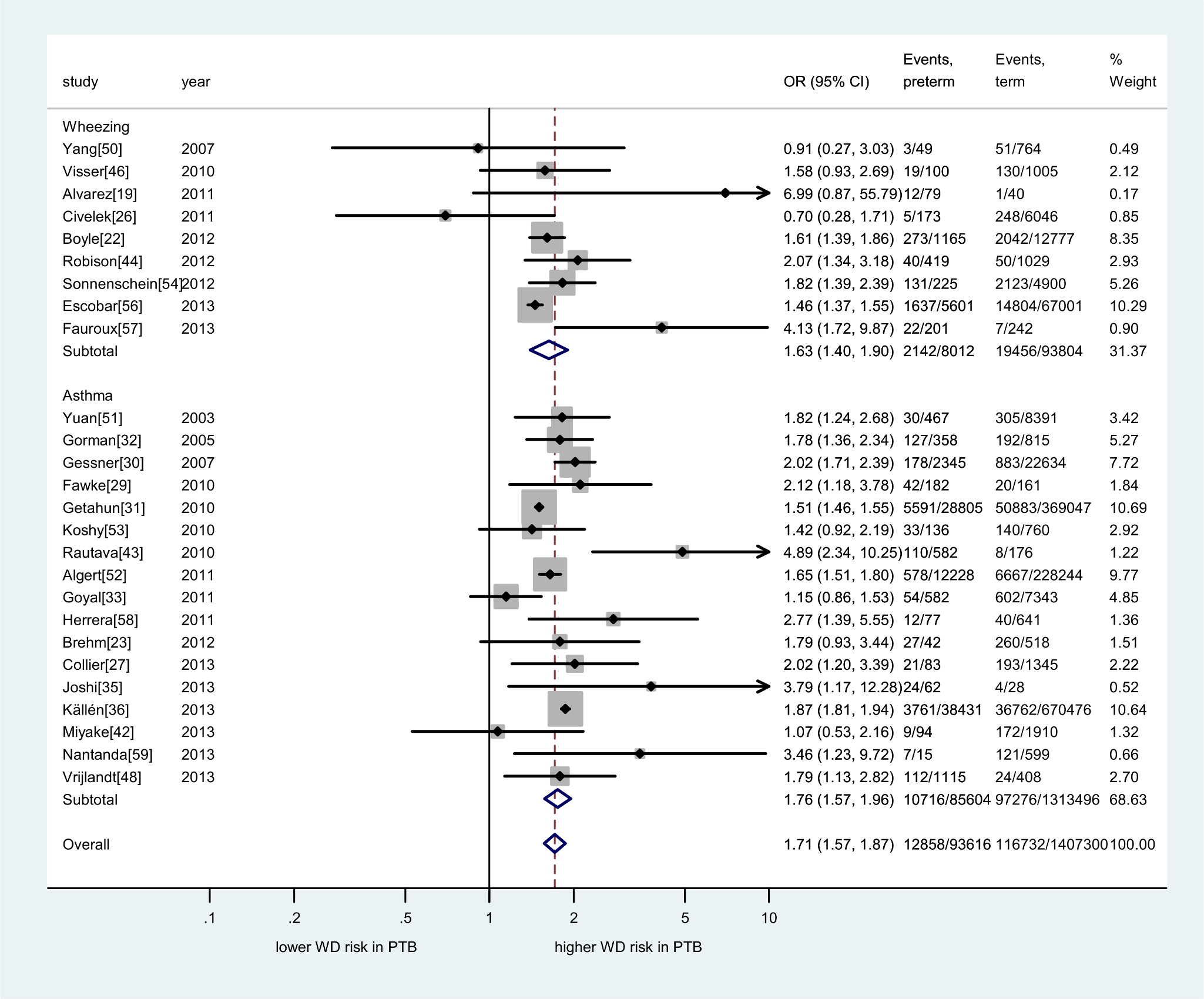

Supplement: Figure S13 — Meta-analysis of unadjusted association between preterm birth and childhood wheezing disorders according to wheezing type. Heterogeneity: I 2 (wheezing) = 52% (95% CI 0%–76%); I 2 (asthma) = 86% (95% CI 79%–90%). PTB, preterm birth; WD, wheezing disorders. (TIF) [file pmed.1001596.s014.tif]

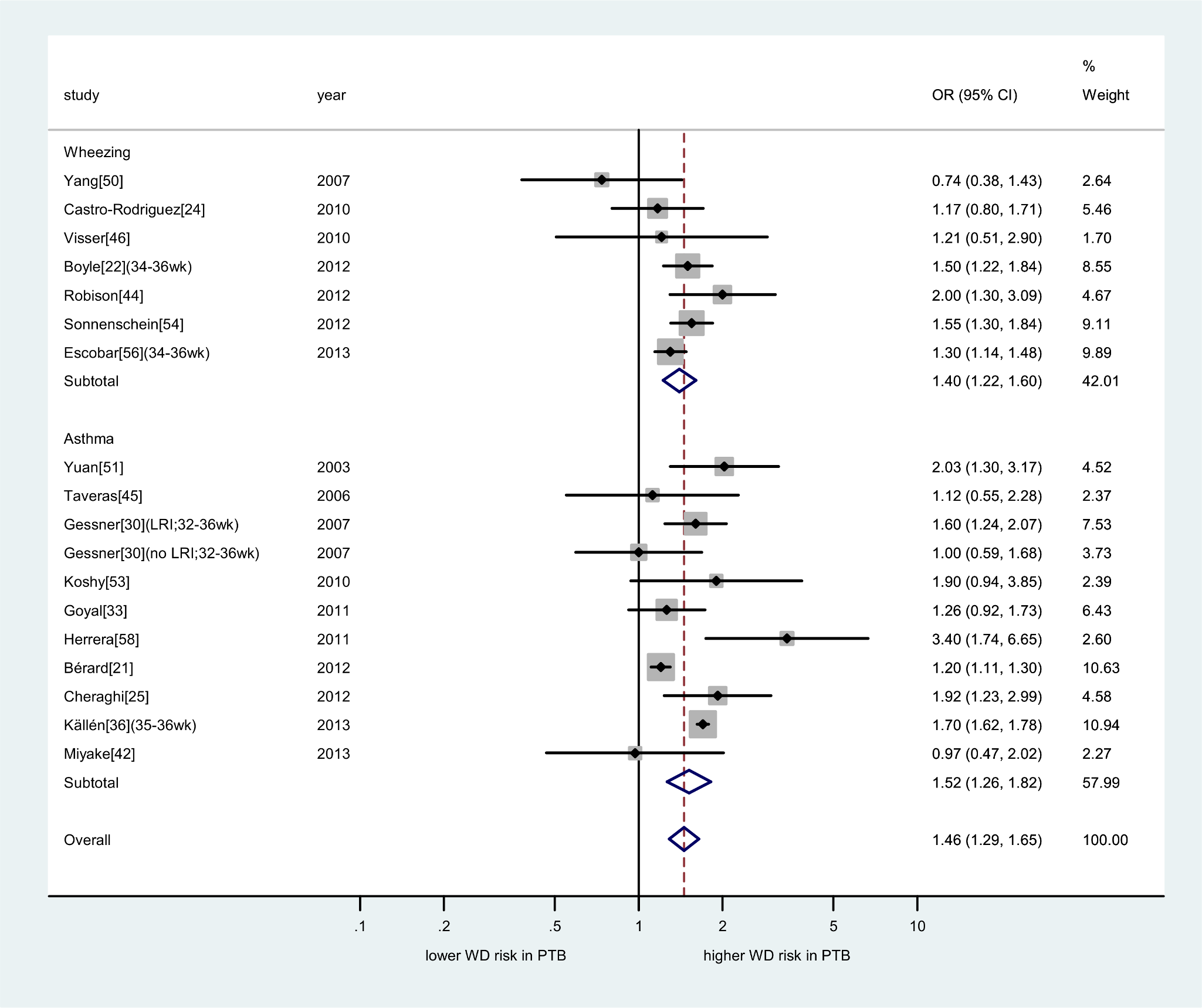

Supplement: Figure S14 — Meta-analysis of adjusted association between preterm birth and childhood wheezing disorders according to wheezing type. Subgroups taken from individual studies noted in parentheses. Confounders adjusted for in individual studies are outlined in Figure 3 and Table S3. Heterogeneity: I 2 (wheezing) = 41% (95% CI 0%–74%); I 2 (asthma) = 86% (95% CI 76%–90%). PTB, preterm birth; WD, wheezing disorders. (TIF) [file pmed.1001596.s015.tif]

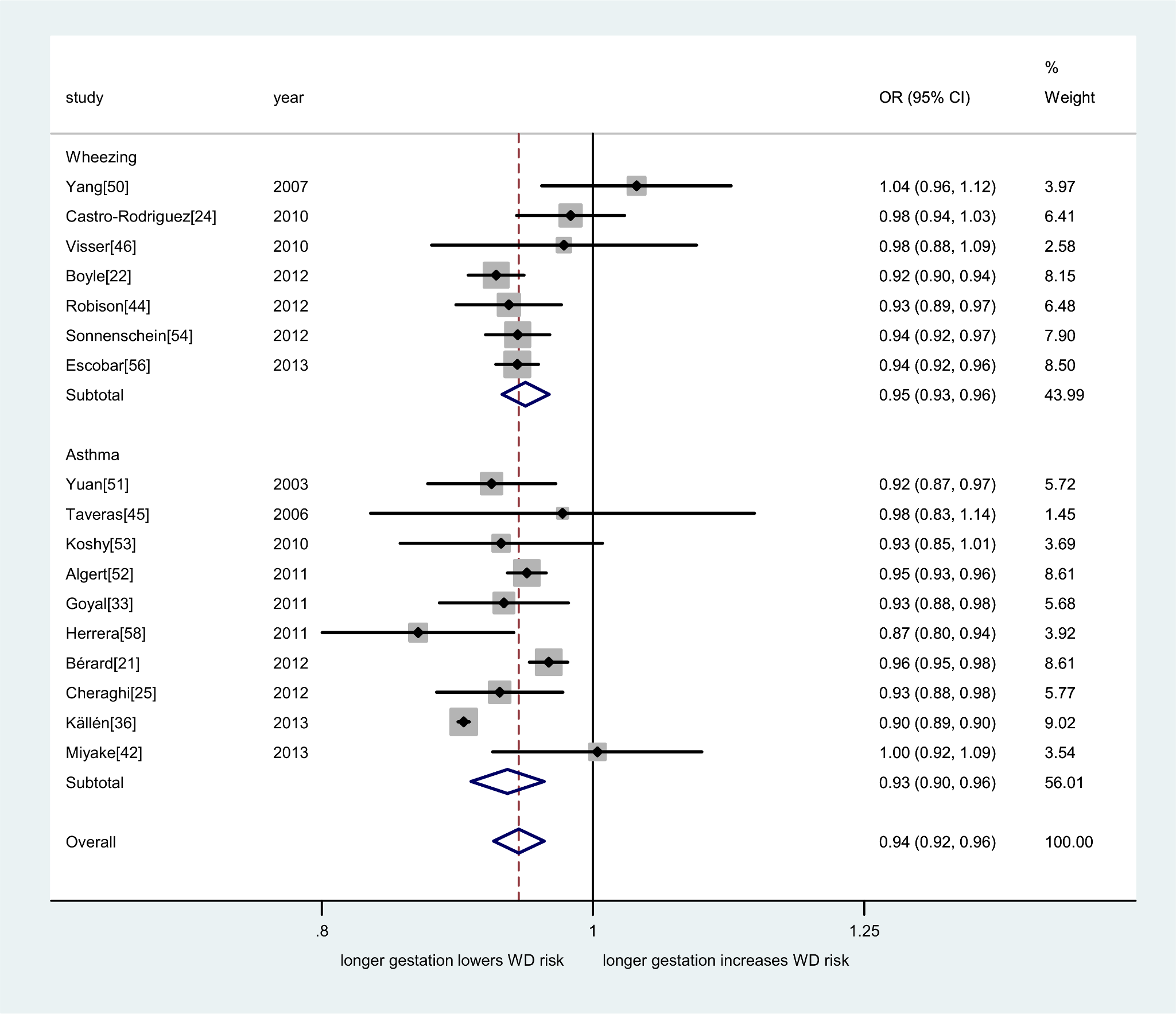

Supplement: Figure S15 — Meta-analysis of adjusted dose–response association between gestational age (per week increase) and childhood wheezing disorders according to wheezing type. Confounders adjusted for in individual studies are outlined in Figure 4 and Table S3. Heterogeneity: I 2 (wheezing) = 52% (95% CI 0%–78%); I 2 (asthma) = 92% (95% CI 87%–94%). WD, wheezing disorders. (TIF) [file pmed.1001596.s016.tif]
