# Supplementary material for: Preterm Birth and Childhood Wheezing Disorders: A Systematic Review and Meta-Analysis
Source: PLoS Med. 2014 Jan 28;11(1):e1001596. doi: 10.1371/journal.pmed.1001596 (PMC3904844; doi:10.1371/journal.pmed.1001596)
Supplement: Table S1 — Characteristics of studies excluded because of population overlap. *Selected inhaled or systemic adrenergics, anticholinergics, xantines, antiallergics, leukotriene receptor antagonists, and/or steroids; ** as compared to preterm babies without BPD. GA, gestational age; KFHP, Kaiser Foundation Health Plan; KPMCP, Kaiser Permanente Medical Care Program; N/R, not reported; LMP, last menstrual period; SD, standard deviation. (DOCX) [file pmed.1001596.s017.docx]

| **Author, yr [ref]** | **Study design** | **Sample size** | **Inclusion criteria** | **Exclusion criteria** | **Birth year** | **Country (region)** | **Age at follow up** | **Exposure ascertainment** | **Outcome**  **definition** | **Outcome ascertainment** | **Unadjusted association** | | **Adjusted association** | |
| --- | --- | --- | --- | --- | --- | --- | --- | --- | --- | --- | --- | --- | --- | --- |
|  |  |  |  |  |  |  |  |  |  |  | **OR** | **95%CI** | **OR** | **95%CI** |
| Alm 2008 [18] | Pro-spective cohort | 4,921 | Random selection of children born in region in 2003 | No response | 2003 | Sweden (Western Sweden) | 1 yr | Unclear | Inhaled corticosteroids | Parental questionnaire | 2.1 | 1.5-3.0 | 1.6 | 1.0-2.6 |
|  | Reasons for exclusion as opposed to Källén 2013 [36]: smaller sample size, shorter follow-up, parental report | | | | | | | | | | | | | |
| Aspberg 2010 [20] | Retro-spective cohort | 1,325,195 | Singleton | Unable to link | 1990-2003 | Sweden | 2-15 yr | Swedish Birth Register | Asthma drug prescription* July-Dec 2005 | Swedish Prescribed Drugs Register | 1.54 | 1.49-1.59 | 1.52 | 1.47-1.57 |
|  | Reasons for exclusion as opposed to Källén 2013 [36]: shorter follow-up, many subjects born before 1995, less detailed gestational age data | | | | | | | | | | | | | |
| Escobar 2010 [28] | Retro-spective cohort | 71,102 | Born at selected KPMCP hospital + KFHP use or membership 1^st^ 5 yrs | In-hospital death, GA <32 wk, incomplete data | 1996-2002 | US (Northern California) | 3 yr | KPMCP hospital records | Recurrent wheeze in 3^re^yr* | ICD-9 code 493.xx or 786.07, or asthma drug prescription in KPMCP hospital records | 1.48 | 1.32-1.67 | 1.23 (34-36 w)  1.40 (32-33w) | 1.07-1.41  1.07-1.82 |
|  | Reason for exclusion as opposed to Escobar 2013 [56]: shorter follow-up, smaller sample size | | | | | | | | | | | | | |
| Hennesy 2008 [34] | Case control | 367 | GA <25 wk; matched term classmate controls | Lost to follow-up | 1995 | UK, Ireland | 5-7 yr | Hospital records | Ever asthma diagnosis | Parental questionnaire | 1.74 | 1.07-2.82 | N/R | N/R |
|  | Reasons for exclusion as opposed to Fawke 2010 [29]: shorter follow-up | | | | | | | | | | | | | |
| Kang 2009 [37] | Pro-spective cohort | 1,500 | Singletons from cohort enriched with asthmatic mothers | Non-English speaking mothers, infant death, no consent, no medical record data | 1997-2000 | US (New England) | 6 yr (± 3 mo) | Unclear | Asthma + wheeze in prior year | Parental reported physician diagnosis (asthma) or symptom (wheezing) | 1.92 | 1.16-3.17 | N/R | N/R |
|  | Reason for exclusion as opposed to Collier 2013 [27]: no crude figures given | | | | | | | | | | | | | |
| Koshy 2013 [53] | Cross-sectional | 857 | Children attending primary school in Merseyside | Non-response | 1995-2001 | UK (Merseyside) | 5-11 yr | Parental questionnaire | Asthma | Parental reported physician diagnosis | 1.55 | 1.00-2.41 | N/R | N/R |
|  | Reason for exclusion as opposed to Koshy 2010 [38]: smaller sample size | | | | | | | | | | | | | |
| Kumar 2008 [39] | Pro-spective cohort | 1,096 | Singletons born in Boston Medical Center | No consent, incomplete data | 1998- 20?? | US (Boston) | 0·5-6 yr | Boston Medical Center hospital records (LMP + 1^st^ trimester ultrasound) | Recurrent (≥ 2 episodes) wheezing | Physician-documented wheezing in Boston Medical Center medical records | 2.15 | 1.50-1.85 | N/R | N/R |
|  | Reason for exclusion as opposed to Robison 2012 [44]: smaller sample size | | | | | | | | | | | | | |
| Kumar 2012 [40] | Pro-spective cohort | 1,034 | Singletons born in Boston Medical Center | No consent, incomplete data | 1998- 20?? | US (Boston) | 0·5-6 yr | Boston Medical Center hospital records (LMP + 1^st^ trimester ultrasound) | Recurrent (≥ 3 episodes) wheezing | Physician-documented wheezing in Boston Medical Center medical records | 2.62 | 1.57-4.39 | 2.36 | 1.39-3.99 |
|  | Reason for exclusion as opposed to Robison 2012 [44]: smaller sample size | | | | | | | | | | | | | |
| Lum 2011 [41] | Case control | 101 | GA <25 wk; matched term classmate controls | Lost to follow-up | 1995 | UK, Ireland | 11 yr | Hospital records | Current symptoms / doctor diagnosis of asthma + medication in last year | Parental questionnaire | 2.32 | 0.84-6.42 | N/R | N/R |
|  | Reasons for exclusion as opposed to Fawke 2010 [29]: smaller sample size | | | | | | | | | | | | | |
| Örtqvist 2009 [55] | Cross sectional | 10,778 | Twins | No consent, unable to link, missing data | 1992-1998 | Sweden | 9-12 yr | Swedish Birth Register | Parental reported doctor diagnosis | Parental questionnaire | 1.58 | 1.42-1.76 | 1.29 (35-36w) 1.70 (32-34w) 2.37 (<32w) | 1.06-1.56 1.36-2.14 1.78-3.17 |
|  | Reasons for exclusion as opposed to Källén 2013 [36]: smaller sample size, selected population | | | | | | | | | | | | | |
| Vogt 2011 [47] | Retro-spective cohort | 254,579 (6-9 yr only) | Born to 2 Swedish parents, living in Sweden on 31 Dec 2005 | Major anomalies, birth weight for gestation >3SD or <−6SD | 1997-2000 | Sweden | 6-9 yr | Swedish Birth Register | Inhaled corticosteroid use in 2006 | Anatomical Therapeutic Chemical Code R03AK or R03BA Swedish Prescribed Drug Register | N/R | N/R | 1.24 (35-36w) 1.35 (33-34w) 1.57 (29-32w) 2.02 (23-28w) | 1.19-1.30 1.25-1.46 1.43-1.72 1.73-2.37 |
|  | Reasons for exclusion as opposed to Källén 2013 [36]: smaller sample size, no crude data provided | | | | | | | | | | | | | |
| Welsh 2009 [49] | Case control | 76 | GA <25 wk; matched term classmate controls | Lost to follow-up | 1995 | UK, Ireland | 11 yr | Hospital records | Current asthma | Parental questionnaire | 2.46 | 0.81-7.46 | N/R | N/R |
|  | Reasons for exclusion as opposed to Fawke 2010 [29]: smaller sample size | | | | | | | | | | | | | |

**Table S1. Characteristics of studies excluded because of population overlap.** *Selected inhaled or systemic adrenergics, anticholinergics, xantines, antiallergics, leukotriene receptor antagonists, and/or steroids; ** as compared to preterms without bronchopulmonary dysplasia; KPMCP: Kaiser Permanente Medical Care Program; KFHP: Kaiser Foundation Health Plan; US: United States; GA: gestational age; UK: United Kingdom; N/R: not reported; LMP: last menstrual period; SD: standard deviation
