# Supplementary material for: Preterm Birth and Childhood Wheezing Disorders: A Systematic Review and Meta-Analysis
Source: PLoS Med. 2014 Jan 28;11(1):e1001596. doi: 10.1371/journal.pmed.1001596 (PMC3904844; doi:10.1371/journal.pmed.1001596)
Supplement: Table S2 — Assessment of study quality. Scoring according to the Effective Public Health Practice Project quality assessment tool for quantitative studies. For study design “7” indicates cross-sectional design in all cases. N/A, not applicable. (DOCX) [file pmed.1001596.s018.docx]

|  | Selection bias | | | Study design | | Confounders | | | Blinding | | | Data collection | | | Withdrawals + dropouts | | | Global  rating | Risk of bias |
| --- | --- | --- | --- | --- | --- | --- | --- | --- | --- | --- | --- | --- | --- | --- | --- | --- | --- | --- | --- |
| Author, yr [ref] | Q1 | Q2 | Rating | Design | Rating | Q1 | Q2 | Rating | Q1 | Q2 | Rating | Q1 | Q2 | Rating | Q1 | Q2 | Rating |  |  |
| Algert 2012 [52] | 1 | 4 | 1 | 5 | 2 | 3 | 4 | 3 | 2 | 2 | 1 | 1 | 1 | 1 | 1 | 1 | 1 | 2 | moderate |
| Álvarez 2011 [19] | 3 | 1 | 3 | 5 | 2 | 1 | 3 | 3 | 3 | 3 | 2 | 3 | 3 | 3 | 4 | 1 | 1 | 3 | high |
| Bérard 2012 [21] | 3 | 4 | 3 | 5 | 2 | 1 | 2 | 2 | 2 | 2 | 1 | 1 | 1 | 1 | 4 | 1 | 1 | 2 | moderate |
| Boyle 2012 [22] | 1 | 1 | 1 | 5 | 2 | 1 | 2 | 2 | 3 | 3 | 2 | 3 | 3 | 3 | 1 | 2 | 2 | 2 | moderate |
| Brehm 2012 [23] | 1 | 1 | 1 | 4 | 2 | 3 | 1 | 1 | 3 | 3 | 2 | 3 | 3 | 3 | 4 | 1 | 1 | 2 | moderate |
| Castro-Rodriguez 2010 [24] | 1 | 2 | 2 | 5 | 2 | 3 | 1 | 1 | 3 | 3 | 2 | 1 | 3 | 2 | 1 | 1 | 1 | 2 | moderate |
| Cheraghi 2012 [25] | 2 | 2 | 2 | 7 | 3 | 3 | 2 | 2 | 3 | 3 | 2 | 1 | 3 | 2 | 4 | 1 | 1 | 2 | moderate |
| Civelek 2011 [26] | 1 | 1 | 1 | 7 | 3 | 3 | 1 | 1 | 3 | 3 | 2 | 1 | 3 | 2 | 4 | 1 | 1 | 2 | moderate |
| Collier 2012 [27] | 3 | 2 | 3 | 5 | 2 | 3 | 4 | 3 | 3 | 3 | 2 | 3 | 3 | 3 | 1 | 2 | 2 | 3 | high |
| Escobar 2013 [56] | 3 | 4 | 3 | 5 | 2 | 1 | 2 | 2 | 2 | 2 | 1 | 1 | 1 | 1 | 1 | 1 | 1 | 2 | moderate |
| Fauroux 2013 [57] | 3 | 1 | 3 | 4 | 2 | 1 | 3 | 3 | 3 | 3 | 2 | 3 | 3 | 3 | 1 | 1 | 1 | 3 | high |
| Fawke 2011 [29] | 3 | 2 | 3 | 7 | 3 | 1 | 4 | 3 | 3 | 3 | 2 | 1 | 3 | 2 | 1 | 1 | 1 | 3 | high |
| Gessner 2007 [30] | 3 | 4 | 3 | 5 | 2 | 3 | 2 | 2 | 2 | 2 | 1 | 1 | 1 | 1 | 1 | 2 | 2 | 2 | moderate |
| Getahun 2010 [31] | 2 | 4 | 2 | 5 | 2 | 3 | 4 | 3 | 2 | 2 | 1 | 1 | 1 | 1 | 1 | 1 | 1 | 2 | moderate |
| Gorman 2005 [32] | 3 | 2 | 3 | 7 | 3 | 1 | 2 | 2 | 3 | 3 | 2 | 3 | 3 | 3 | 4 | 1 | 1 | 3 | high |
| Goyal 2011 [33] | 3 | 4 | 3 | 5 | 2 | 1 | 3 | 3 | 2 | 2 | 1 | 1 | 1 | 1 | 1 | 1 | 1 | 3 | high |
| Herrera 2011 [58] | 4 | 1 | 3 | 7 | 3 | 3 | 3 | 3 | 3 | 3 | 2 | 1 | 3 | 2 | 4 | 1 | 1 | 3 | high |
| Joshi 2013 [35] | 3 | 5 | 3 | 7 | 3 | 1 | 3 | 3 | 3 | 3 | 2 | 1 | 3 | 2 | 4 | 1 | 1 | 3 | high |
| Källén 2013 [36] | 1 | 4 | 1 | 5 | 2 | 1 | 2 | 2 | 2 | 2 | 1 | 1 | 1 | 1 | 4 | 1 | 1 | 1 | low |
| Koshy 2010 [53] | 3 | 3 | 3 | 7 | 3 | 3 | 3 | 3 | 3 | 3 | 2 | 1 | 3 | 2 | 4 | 1 | 1 | 3 | high |
| Miyake 2013 [42] | 2 | 3 | 3 | 7 | 3 | 3 | 1 | 1 | 3 | 3 | 2 | 1 | 3 | 2 | 4 | 1 | 1 | 3 | high |
| Nantanda 2013 [59] | 3 | 2 | 3 | 7 | 3 | 3 | 3 | 3 | 3 | 3 | 2 | 3 | 3 | 3 | 4 | 1 | 1 | 3 | high |
| Rautava 2010 [43] | 3 | 4 | 3 | 5 | 2 | 3 | 3 | 3 | 2 | 2 | 1 | 1 | 1 | 1 | 4 | 1 | 1 | 3 | high |
| Robison 2012 [44] | 2 | 1 | 2 | 5 | 2 | 3 | 1 | 1 | 3 | 3 | 2 | 1 | 1 | 1 | 1 | 1 | 1 | 1 | low |
| Sonnenschein-van der Voort 2012 [54] | 2 | 2 | 2 | 5 | 2 | 3 | 1 | 1 | 3 | 3 | 2 | 1 | 3 | 2 | 1 | 1 | 1 | 1 | low |
| Taveras 2006 [45] | 3 | 2 | 3 | 5 | 2 | 3 | 2 | 2 | 3 | 3 | 2 | 3 | 3 | 3 | 1 | 2 | 2 | 3 | high |
| Visser 2010 [46] | 1 | 1 | 1 | 7 | 3 | 3 | 1 | 1 | 2 | 3 | 2 | 1 | 3 | 2 | 4 | 1 | 1 | 2 | moderate |
| Vrijlandt 2013 [48] | 3 | 2 | 3 | 5 | 2 | 1 | 3 | 3 | 3 | 3 | 2 | 1 | 3 | 2 | 1 | 2 | 2 | 3 | high |
| Yang 2007 [50] | 2 | 5 | 2 | 5 | 2 | 3 | 2 | 2 | 3 | 3 | 2 | 3 | 3 | 3 | 1 | 2 | 2 | 2 | moderate |
| Yuan 2003 [51] | 1 | 4 | 1 | 5 | 2 | 3 | 1 | 1 | 2 | 2 | 1 | 1 | 1 | 1 | 1 | 1 | 1 | 1 | low |

**Table S2. Assessment of study quality.** Scoring according to the Effective Public Health Practice Project quality assessment tool for quantitative studies. For study design ‘7’ indicates cross-sectional design in all cases. N/A = not applicable.
