# Supplementary material for: Preterm Birth and Childhood Wheezing Disorders: A Systematic Review and Meta-Analysis
Source: PLoS Med. 2014 Jan 28;11(1):e1001596. doi: 10.1371/journal.pmed.1001596 (PMC3904844; doi:10.1371/journal.pmed.1001596)
Supplement: Table S3 — Covariates in multivariate models of individual studies associating preterm birth with wheezing disorders. amonth of conception, maternal urinary tract infection, pre-labour rupture of membranes, prostaglandin induction, neonatal jaundice, neonatal sepsis; bthreatened miscarriage, infections, asphyxia, birth trauma, premature rupture of membranes, umbilical cord knots, placenta problems; cCC10 G+38A polymorphisms, cord blood IgE. (DOCX) [file pmed.1001596.s019.docx]

|  | General characteristics | | | | | | | | | | Pregnancy exposures | | | | | | | | | Perinatal exposures | | | | Postnatal exposures | | | | | | | | |
| --- | --- | --- | --- | --- | --- | --- | --- | --- | --- | --- | --- | --- | --- | --- | --- | --- | --- | --- | --- | --- | --- | --- | --- | --- | --- | --- | --- | --- | --- | --- | --- | --- |
| Author, yr [ref] | Age | Sex | Ethnicity | Paternal atopy / asthma | Maternal age | Maternal marital status | Parental education / occupation | Insurance | Income / socio-economic status | Location | Maternal Body Mass Index | Maternal diabetes | Maternal atopy / asthma | Maternal hypertension | Maternal smoking | Maternal alcohol intake | Maternal diet | Multiple exposures | Multiple birth | Delivery method | (Low) birth weight | Congenital anomalies | Neonatal lung disease | Breastfeeding | Smoke exposure | Parity / siblings | Day care attendance | Housing conditions | Cockroaches / pets | Atopy | Respiratory infections / snoring | Obesity / overweight |
| Algert 2011 [52] | x |  |  | x | x |  |  |  | x | x |  | x |  | x | x |  |  | x^a^ |  | x | x |  | x |  |  | x |  |  |  |  |  |  |
| Boyle 2010 [22] | x | x | x |  | x | x | x |  |  |  |  |  |  |  | x | x |  |  |  |  |  |  |  | x |  | x |  |  |  |  |  |  |
| Bérard 2012 [21] |  | x |  |  | x | x | x | x |  | x |  | x | x | x |  |  |  |  |  |  | x |  |  |  |  |  |  |  |  |  |  |  |
| Castro-Rodriguez 2010 [24] |  | x |  |  |  |  |  |  |  |  |  |  | x |  | x |  | x |  |  |  |  |  |  | x |  | x | x | x |  | x |  |  |
| Cheraghi 2012 [25] |  | x |  | x |  |  |  |  |  | x |  |  |  |  |  |  |  |  |  | x |  |  |  | x | x |  |  | x | x |  | x |  |
| Escobar 2013 [56] |  | x | x | x | x |  |  |  |  |  |  |  | x |  |  |  |  |  |  |  | x | x | x |  |  | x |  |  |  |  | x |  |
| Gessner 2007 [30] | x | x | x |  | x |  | x | x |  | x |  |  |  |  | x |  |  |  | x | x |  |  |  |  |  |  |  |  |  |  |  |  |
| Goyal 2011 [33] |  | x | x |  |  |  |  | x | x | x |  |  |  |  |  |  |  |  |  | x |  |  |  | x |  |  |  |  |  | x |  |  |
| Herrera 2011 [58] |  |  |  | x |  |  |  |  |  |  |  |  | x |  |  |  |  |  |  |  |  |  |  |  |  |  |  | x | x | x | x |  |
| Källén 2013 [36] | x | x |  |  | x |  |  |  |  |  | x |  |  |  | x |  |  |  |  |  |  |  |  |  |  | x |  |  |  |  |  |  |
| Koshy 2010 [53] | x | x |  |  |  |  |  |  | x |  |  |  |  |  |  |  |  |  |  |  | x |  |  |  |  |  |  |  |  | x |  | x |
| Miyake 2013 [42] |  | x |  | x |  |  | x |  |  |  |  |  | x |  | x |  |  |  |  |  |  |  |  | x | x | x |  |  |  |  |  |  |
| Robison 2012 [44] | x | x | x |  |  |  |  |  | x |  |  |  | x |  | x |  |  |  |  |  |  |  |  | x | x |  |  |  |  |  |  |  |
| Sonnenschein-van der Voort 2012 [54] |  | x | x |  | x |  | x |  |  |  | x | x | x | x | x |  |  |  |  |  |  |  |  | x |  | x | x |  | x |  |  |  |
| Taveras 2006 [45] |  | x |  | x | x |  |  |  |  |  | x |  | x |  |  |  |  |  |  |  |  |  |  | x | x | x |  |  |  |  |  |  |
| Visser 2010 [46] |  | x |  | x |  |  |  |  |  |  |  | x | x |  | x |  |  | x^b^ |  | x | x |  |  |  |  | x | x | x |  | x |  |  |
| Yang 2007 [50] |  | x |  | x |  |  |  |  |  |  |  |  |  |  |  |  |  | x^c^ |  |  |  |  |  |  | x |  |  |  |  |  |  |  |
| Yuan 2003 [51] |  | x |  |  | x | x |  |  |  |  |  |  | x |  | x |  |  |  |  |  |  |  |  |  |  | x |  |  |  |  |  |  |

**Table S3. Covariates in multivariate models of individual studies associating preterm birth with wheezing disorders.**^a^ month of conception, maternal urinary tract infection, pre-labour rupture of membranes, prostaglandin induction, neonatal jaundice, neonatal sepsis; ^b^ threatened miscarriage, infections, asphyxia, birth trauma, premature rupture of membranes, umbilical cord knots, placenta problems; ^c^ CC10 G+38A polymorphisms, cord blood IgE.
