# Supplementary material for: Preterm Birth and Childhood Wheezing Disorders: A Systematic Review and Meta-Analysis
Source: PLoS Med. 2014 Jan 28;11(1):e1001596. doi: 10.1371/journal.pmed.1001596 (PMC3904844; doi:10.1371/journal.pmed.1001596)
Supplement: Text S1 — PROSPERO-registered review protocol. (PDF) [file pmed.1001596.s020.pdf]

PROSPERO International prospective register of systematic reviews

Review title and timescale

1 Review title
Give the working title of the review. This must be in English. Ideally it should state succinctly the interventions or exposures being reviewed and the associated health or social problem being addressed in the review.
Preterm birth as a risk factor for childhood wheezing disorders: a systematic review and meta-analysis

2 Original language title
For reviews in languages other than English, this field should be used to enter the title in the language of the review. This will be displayed together with the English language title.

3 Anticipated or actual start date
Give the date when the systematic review commenced, or is expected to commence.
01/04/2013

4 Anticipated completion date
Give the date by which the review is expected to be completed.
01/08/2013

5 Stage of review at time of this submission
Indicate the stage of progress of the review by ticking the relevant boxes. Reviews that have progressed beyond the point of completing data extraction at the time of initial registration are not eligible for inclusion in PROSPERO. This field should be updated when any amendments are made to a published record.

The review has not yet started
No

Review stage	Started	Completed
Preliminary searches	No	Yes
Piloting of the study selection process	No	Yes
Formal screening of search results against eligibility criteria	No	Yes
Data extraction	Yes	No
Risk of bias (quality) assessment	Yes	No
Data analysis	Yes	No

Provide any other relevant information about the stage of the review here.
Funding obtained. Currently in data-extraction process.

Review team details

6 Named contact
The named contact acts as the guarantor for the accuracy of the information presented in the register record.
Jasper Been

7 Named contact email
Enter the electronic mail address of the named contact.
jasperbeen@gmail.com

8 Named contact address
Enter the full postal address for the named contact.
Teviot Place doorway 3 Edinburgh EH8 9AG United Kingdom

9 Named contact phone number
Enter the telephone number for the named contact, including international dialing code.
00441316509232

10 Organisational affiliation of the review
Full title of the organisational affiliations for this review, and website address if available. This field may be completed as 'None' if the review is not affiliated to any organisation.

School for Public Health and Primary Care (CAPHRI), Maastricht University

Website address:
www.caphri.nl

11 Review team members and their organisational affiliations
Give the title, first name and last name of all members of the team working directly on the review. Give the organisational affiliations of each member of the review team.

Title	First name	Last name	Affiliation
Dr	Jasper	Been	School for Public Health and Primary Care (CAPHRI), Maastricht University, Maastricht, Netherlands
Miss	Marlies	Lugtenberg	School for Public Health and Primary Care (CAPHRI), Maastricht University, Maastricht, Netherlands
Miss	Eline	Smets	School for Public Health and Primary Care (CAPHRI), Maastricht University, Maastricht, Netherlands
Professor	Constant	van Schayck	School for Public Health and Primary Care (CAPHRI), Maastricht University, Maastricht, Netherlands
Professor	Boris	Kramer	Department of Paediatrics, Maastricht University Medical Centre, Maastricht, Netherlands
Dr	Monique	Mommers	School for Public Health and Primary Care (CAPHRI), Maastricht University, Maastricht, Netherlands
Professor	Aziz	Sheikh	Centre for Population Health Sciences, The University of Edinburgh, Edinburgh, United Kingdom

12 Funding sources/sponsors
Give details of the individuals, organizations, groups or other legal entities who take responsibility for initiating, managing, sponsoring and/or financing the review. Any unique identification numbers assigned to the review by the individuals or bodies listed should be included.
JB is supported by a Maastricht University Kootstra Talent Fellowship JB is supported by the International Pediatric Research Foundation Young Investigators Exchange Programme

13 Conflicts of interest
List any conditions that could lead to actual or perceived undue influence on judgements concerning the main topic investigated in the review.
Are there any actual or potential conflicts of interest?
None known

14 Collaborators
Give the name, affiliation and role of any individuals or organisations who are working on the review but who are not listed as review team members.

Title	First name	Last name	Organisation details

Review methods

15 Review question(s)
State the question(s) to be addressed / review objectives. Please complete a separate box for each question.
Is preterm birth associated with the development of wheezing disorders during childhood?

16 Searches
Give details of the sources to be searched, and any restrictions (e.g. language or publication period). The full search

strategy is not required, but may be supplied as a link or attachment.
Two reviewers will independently search PubMed, EMBASE, TRIP database, and Google Scholar for eligible studies. We will identify additional studies by screening reference lists and citations of articles of interest via ISI Web of Knowledge. We will also approach an international panel of experts to identify ongoing and unpublished studies, as well as studies that might have been missed by our search strategy.

17 URL to search strategy
If you have one, give the link to your search strategy here. Alternatively you can e-mail this to PROSPERO and we will store and link to it.

18 Condition or domain being studied
Give a short description of the disease, condition or healthcare domain being studied. This could include health and wellbeing outcomes.
Wheezing disorders during childhood, defined as a diagnosis or reported symptoms of wheezing or asthma.

19 Participants/population
Give summary criteria for the participants or populations being studied by the review. The preferred format includes details of both inclusion and exclusion criteria.
Children aged 0.5-18 years. Only studies where at least 50% of the cohort is born from 1995 onwards, and none of the children is born before 1990, are eligible. The rationale for this is that important practice changes in neonatal medicine have contributed to improved outcomes among (very) preterm infants in the last two decades. To obtain an estimate that is relevant to current neonatal practice, we have set this pragmatic time constraint.

20 Intervention(s), exposure(s)
Give full and clear descriptions of the nature of the interventions or the exposures to be reviewed
The exposure under study is preterm birth, defined as being born at a gestational age less than 37 completed weeks. Studies comparing the outcome between one or more subgroups at the more extreme end of gestation (e.g. =37 weeks). Studies using broader gestational age definitions to define term births (e.g. >= 36 weeks) will not be considered. Post-term births (>42 weeks) will be excluded when possible.

22 Types of study to be included initially
Give details of the study designs to be included in the review. If there are no restrictions on the types of study design eligible for inclusion, this should be stated.
We will consider the following observational designs for inclusion: cohort, case-control, and cross-sectional studies. We do not expect to identify experimental or quasi-experimental studies relevant to the research question. However if they are identified we will consider their inclusion if relevant. Two reviewers will independently assess study eligibility. The final decision to include will be based on consensus and arbitration by a third author if necessary. When studies have significantly overlapping populations, one study will be selected for final inclusion, study size being taken into account as a major determinant.

23 Context
Give summary details of the setting and other relevant characteristics which help define the inclusion or exclusion criteria.

24 Primary outcome(s)
Give the most important outcomes.
The primary outcome is any wheezing disorder. We will use the following hierarchy to select the outcome from studies reporting multiple outcomes: asthma; persistent wheezing; recurrent wheezing; severe wheezing; and wheezing. We will use the following hierarchy to select the outcome with the highest level of ascertainment: clinician diagnosis; documented medication use as a wheezing disorders proxy; routinely collected health care data; parental or patient-reported physician diagnosis; parental or patient-reported medication use; and parental or patient-reported symptoms. ‘Ever’ wheezing/asthma will be favoured over recent and current wheezing disorders, respectively.

Give information on timing and effect measures, as appropriate.

25 Secondary outcomes
List any additional outcomes that will be addressed. If there are no secondary outcomes enter None.
None.

Give information on timing and effect measures, as appropriate.

26 Data extraction, (selection and coding)

Give the procedure for selecting studies for the review and extracting data, including the number of researchers involved and how discrepancies will be resolved. List the data to be extracted.

Two reviewers will independently extract the following data from selected studies:authors; full reference; study design; study location; sample size; inclusion- and exclusion criteria; age range and birth year of study participants; method of ascertainment of exposure and outcomes; outcome measure; unadjusted and adjusted association measures. Unadjusted association measures will preferably be calculated using crude data via production of 2x2 tables. If available, adjusted association estimates of a linear relationship between gestational age and wheezing disorder risk will be extracted to assess a possible “dose-response relationship” between degree of prematurity and risk of wheezing disorders.

27 Risk of bias (quality) assessment

State whether and how risk of bias will be assessed, how the quality of individual studies will be assessed, and whether and how this will influence the planned synthesis.

Two reviewers will independently assess risk of bias for each included study using the Effective Public Health Practice Project quality assessment tool for quantitative studies. For this purpose the following covariates are considered to be the main confounders of the association between preterm birth and wheezing disorders: gender; maternal smoking during pregnancy; maternal atopy or asthma, or a family history of atopy or asthma. Each study will be assigned to one of the following categories: low risk of bias, moderate risk of bias, and high risk of bias. Again, final judgment of risk of bias will be based on consensus with arbitration by a third author if necessary.

28 Strategy for data synthesis

Give the planned general approach to be used, for example whether the data to be used will be aggregate or at the level of individual participants, and whether a quantitative or narrative (descriptive) synthesis is planned. Where appropriate a brief outline of analytic approach should be given.

Unadjusted association measures will be pooled via Mantel-Haenszel analysis. We anticipate important heterogeneity among the studies and will perform all analyses using a random effects model. Adjusted effect estimates will be pooled using generic inverse variance. Standard errors for the individual study point estimates will be calculated from the respective confidence intervals as described in the Cochrane Handbook (chapter 7). When studies report adjusted association measures for several gestational age strata, the least preterm stratum will be selected for the comparison so as to arrive at the most conservative estimate. Adjusted effect estimates of a dose-response relationship between gestational age and wheezing disorders will be calculated for individual studies that reported multiple gestational age strata using a fixed-effects log-linear dose-response regression model. Individual study summary estimates will then be pooled using generic inverse variance. Between-study heterogeneity will be assessed using Q-statistic and the I-squared test. We will perform meta-regression analysis using the following study characteristics to assess their contribution to overall study heterogeneity: study size, population age, diagnosis ascertainment, wheezing type, and publication year. Small study effects will be assessed using funnel plots, and tested using Harbord’s test for unadjusted association measures, and Egger’s test for adjusted association measures. If possible, population attributable risks will be calculated using the data as described by Rockhill et al (Am J Public Health 1998). All analyses will be performed using Stata 12 software.

29
