# Supplementary material for: Preterm Birth and Childhood Wheezing Disorders: A Systematic Review and Meta-Analysis
Source: PLoS Med. 2014 Jan 28;11(1):e1001596. doi: 10.1371/journal.pmed.1001596 (PMC3904844; doi:10.1371/journal.pmed.1001596)
Supplement: Text S2 — Expert panel. (DOCX) [file pmed.1001596.s021.docx]

**Supporting Text S2**

**Expert panel approached**

Professor Eugenio Baraldi, University of Padua, Italy

Professor Andrew Bush, Imperial College London, UK

Professor Adnan Custovic, University of Manchester, UK

Dr. Liesbeth Duijts, Erasmus University Rotterdam, Netherlands

Professor Bengt Källén, University of Lund, Sweden

Professor Jouni Jaakkola, University of Oulu, Finland

Professor Maritta Jaakkola, University of Oulu, Finland

Professor Sailesh Kotecha, University of Cardiff, UK

Dr. Rajesh Kumar, Children’s Memorial Hospital Chicago, USA

Professor Erika von Mutius, Munich University, Germany

Professor Janet Stocks, University College London, UK

Professor David Strachan, St George’s University of London, UK

Professor Xiaobin Wang, Northwestern University, USA

Professor Scott Weiss, Harvard University Boston, USA
